# Discovery of differentially expressed proteins for CAR-T therapy of ovarian cancers with a bioinformatics analysis

**DOI:** 10.18632/aging.206024

**Published:** 2024-07-18

**Authors:** Dito Anurogo, Chao-Lien Liu, Yu-Chu Chang, Yu-Hsiang Chang, J. Timothy Qiu

**Affiliations:** 1 International Ph.D. Program in Cell Therapy and Regenerative Medicine, College of Medicine, Taipei Medical University, Taipei 11031, Taiwan; 2 Faculty of Medicine and Health Sciences, Universitas Muhammadiyah Makassar, Makassar 90221, Indonesia; 3 School of Medical Laboratory Science and Biotechnology, College of Medical Science and Technology, Taipei Medical University, Taipei 11031, Taiwan; 4 PhD Program in Medical Biotechnology, College of Medical Science and Technology, Taipei Medical University, Taipei 11031, Taiwan; 5 Department of Obstetrics and Gynecology, Taipei Medical University Hospital, Taipei 11031, Taiwan; 6 Department of Obstetrics and Gynecology, School of Medicine, College of Medicine, Taipei Medical University, Taipei 110301, Taiwan

**Keywords:** chimeric antigen receptor (CAR)-T cell, ovarian cancer, differentially expressed gene (DEG), protein-protein interaction (PPI) network, CAR-T-cell antigen

## Abstract

Target antigens are crucial for developing chimeric antigen receptor (CAR)-T cells, but their application to ovarian cancers is limited. This study aimed to identify potential genes as CAR-T-cell antigen candidates for ovarian cancers. A differential gene expression analysis was performed on ovarian cancer samples from four datasets obtained from the GEO datasets. Functional annotation, pathway analysis, protein localization, and gene expression analysis were conducted using various datasets and tools. An oncogenicity analysis and network analysis were also performed. In total, 153 differentially expressed genes were identified in ovarian cancer samples, with 60 differentially expressed genes expressing plasma membrane proteins suitable for CAR-T-cell antigens. Among them, 21 plasma membrane proteins were predicted to be oncogenes in ovarian cancers, with nine proteins playing crucial roles in the network. Key genes identified in the oncogenic pathways of ovarian cancers included *MUC1*, *CXCR4*, *EPCAM*, *RACGAP1*, *UBE2C*, *PRAME*, *SORT1*, *JUP*, and *CLDN3*, suggesting them as recommended antigens for CAR-T-cell therapy for ovarian cancers. This study sheds light on potential targets for immunotherapy in ovarian cancers.

## INTRODUCTION

A chimeric antigen receptor for T cells was developed by Zelig Eshhar in 1993 before being developed to increase its effectiveness and potential as an anti-tumor therapy [1]. One of the most influential and specific methods of immune therapy is chimeric antigen receptor (CAR)-T cell therapies. CAR-T-cell therapies have emerged at the forefront of these advancements, demonstrating remarkable potential in transforming cancer treatment paradigms [2]. CAR-T-cell therapy has emerged as a promising immunotherapeutic strategy for managing various malignancies. These engineered T cells are designed to recognize and destroy cancer cells expressing specific antigens, bypassing major histocompatibility complex (MHC) restrictions associated with conventional T-cell activation. Despite the success of CAR-T cell therapy in hematologic malignancies, its potential in solid tumors, such as ovarian cancer (OC), is yet to be fully realized. Key challenges include the identification of target antigens that are highly and specifically expressed by tumor cells but that spare normal tissues.

OC remains a critical challenge in gynecological oncology and is responsible for a significant source of morbidity and mortality for women worldwide. A diagnosis of OC often occurs in advanced stages of the disease due to its non-specific symptoms and the lack of effective early detection methods. The prevalence of OC ranges from 9.2~12.0 per 100,000 women worldwide [3]. Although the median age for diagnosis is 50~79 years, an older age increases the risk of suffering from more aggressive types of OC [4]. Conventional treatment strategies, such as surgery and platinum-based chemotherapy, provide limited improvement in the overall survival (OS) rate, and drug resistance may be induced [5, 6], underscoring the urgent need for novel therapeutic interventions. Genetically engineered immune effector cells, programmed to recognize specific antigens, offer a ground-breaking approach to target and eliminate malignant cells selectively. Identifying target antigens, particularly for specific cancer types, remains a formidable task, yet it is critical to the success of CAR-T-cell therapies [7].

The potential deployment of CAR-T-cell therapies in OC is increasingly recognized; however, our understanding of suitable antigenic targets remains in its infancy. Addressing this knowledge gap will significantly propel the development of more effective, personalized CAR-T-cell therapies for OC. In this era of precision medicine, the utility of bioinformatics cannot be overstated. Bioinformatics, the application of computational tools to manage, analyze, and interpret biological data, has become a mainstay in cancer research, particularly in the era of high-throughput genomics. Integrating genomic, transcriptomic, and proteomic data from various sources can provide a comprehensive landscape of tumor biology, facilitating the discovery of potential targets for novel therapeutics [8]. In this study, we aimed to leverage the power of bioinformatics to uncover potential targets for CAR-T-cell therapy in the context of OC. By interrogating genomic, transcriptomic, and proteomic datasets, we sought to identify preferentially and highly expressed antigens in ovarian tumor tissues. The bioinformatics strategy we employed offers an accelerated, systematic, and unbiased approach to antigen discovery. We hypothesized that such a comprehensive analysis could propel significant advancements in CAR-T-cell-based immunotherapeutic interventions for OC.

We remain aware of the challenges ahead and are optimistic about the potentially transformative impact that a successful CAR-T-cell therapy could have on the management of OC. Identifying novel targets would not only serve to broaden the therapeutic arsenal. Still, it would also contribute to a deeper understanding of OC biology, paving the way for personalized and precision medicine for this intractable disease.

## RESULTS

### Identification and functional examination of differentially expressed genes (DEGs)

**Figure 1:**
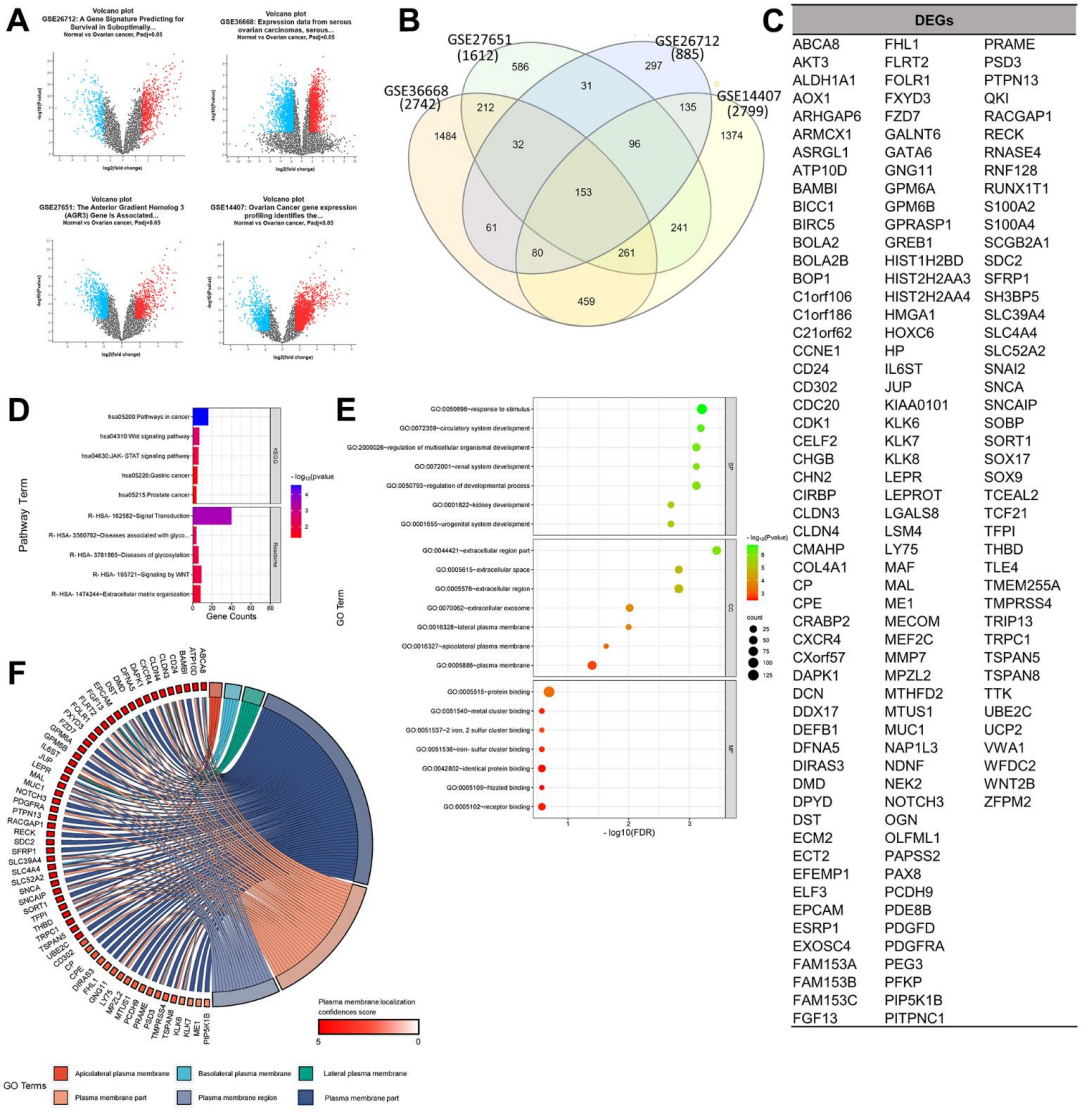
**Determination of gene set enrichments (GSEs) and differentially expressed genes (DEGs).** (**A**) Volcano plot of gene distributions in control and ovarian cancer samples. Gray dots represent genes that are not significantly expressed between normal and ovarian cancer cell samples. (**B**) Venn diagram of overlapping gene between four GSEs from which we obtained 153 DEGs. Also, the highest unique genes are GSE36668, GSE14407, and GSE27651. (**C**) List of DEGs. (**D**) Pathway terms related to DEGs, colored by –log10(*p*-values). (**E**) Gene ontology terms related to DEGs, colored by –log10 (*p*-values). (**F**) Plasma membrane-related genes from six GO terms (*p* > 0.05) with localization confidence scores. A higher score means a greater probability that the protein will be situated there.

The selected gene set enrichments (GSEs), namely GSE36668 [9], GSE27651 [10], GSE26712 [11, 12], and GSE14407 [13], were microarray datasets from OC patient samples, and a detailed profile of the datasets is provided in Table 1. Based on a log [fold change (FC)] cutoff of > 1.5 and a *p*-value of < 0.5, the gene distribution of the sample is clearly visible on the left and right of the volcano plot to ensure the significance of their expressions in the sample. Based on a Venn analysis, it was found that the numbers of unique genes from the GSEs were sequentially 1484, 586, 297, and 1374 genes, and 153 genes, which were DEGs, were expressed in all four GSEs (Figure 1A–1C). Furthermore, results of the gene ontology (GO) term and pathway analysis of DEGs showed a high confidence score (false discovery rate; FDR) for GO biological processes (BPs) and cellular components (CCs) and a low confidence score for GO molecular functions (MFs). GO terms that involved the most GSEs were GO:0005515-protein binding (125 genes: *p* < 0.001), GO:0050896-response to stimulus (102 genes: *p* < 0.0001), and GO:0005886-plasma membrane (60 genes: *p* < 0.001). Based on the results of this analysis, it can be seen that expressed proteins were dominated by those located in plasma membranes and played roles in responding to stimuli, including binding between proteins. Furthermore, the pathway analysis of DEGs showed a relatively low average confidence score (*p*-value). The induced pathways with the highest confidence scores (*p* < 0.0001) were hsa05200: Pathways in Cancer (KEGG: 16 genes) and R-HSA-162582: Signal Transduction (Reactome: 40 genes). Based on the top pathway that was induced in general, it is known that several DEGs act as oncogenes, which can be seen from the term-related pathways to cancer, especially in the Wnt and Janus kinase (JAK)-signal transduction and activator of transcription (STAT) signaling pathways (Figure 1D, 1E).

**Table 1:** Ovarian cancer patient gene expression omnibus (GEO) microarray profiles.

**GEO profile**	**Type**	**Source**	**No. of cases**	**No. of controls**	**Platform**	**Annotation platform**
GSE36668	mRNA	Serous ovarian carcinoma	8	4	GPL570	[HG-U133_Plus_2] Affymetrix Human Genome U133 Plus 2.0 Array
GSE27651	mRNA	High-grade serous ovarian carcinomas	22	6	GPL570	[HG-U133_Plus_2] Affymetrix Human Genome U133 Plus 2.0 Array
GSE26712	mRNA	Ovarian cancer	185	10	GPL96	[HG-U133A] Affymetrix Human Genome U133A Array
GSE14407	mRNA	Serous ovarian cancer epithelia	12	12	GPL570	[HG-U133_Plus_2] Affymetrix Human Genome U133 Plus 2.0 Array

### Prediction of oncogenicity of plasma membrane-related genes as CAR antigens

**Figure 2:**
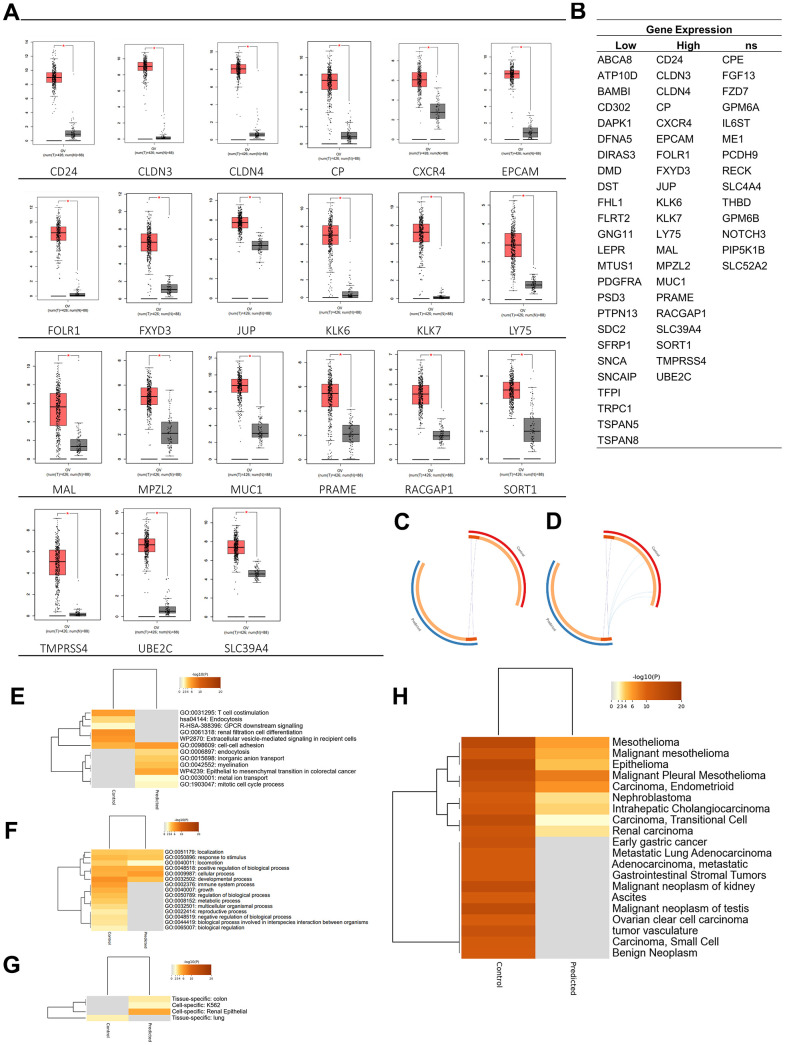
**Oncogenicity analysis.** (**A**) Twenty-one genes were significantly expressed in ovarian cancer among plasma membrane-related genes. (**B**) Plasma membrane-related genes expressions in ovarian cancer. (**C**) Overlap between gene lists: high expression gene list (Predicted) and Control gene list, (**D**) including the shared term level, where blue curves connect genes that share an enriched ontology term. Gene lists are represented by the inner circle, with hits arranged along the arc. Genes that appear on multiple lists are depicted in dark orange, while genes that only appear on one list are displayed in light orange. (**E**) Heatmap of enriched terms across input gene lists, colored by *p*-values. (**F**) Heatmap of biological processes across input gene lists, colored by *p*-values. (**G**) Pattern genes related to predicted and control genes. (**H**) DisGeNET is a discovery platform containing one of the largest publicly available collections of genes and variants associated with human diseases. The heatmap is colored by *p*-values. Dark orange indicates a greater probability that bioactivity will occur.

In the analysis of target antigen CARs, in addition to significant oncogenicity, the protein must be located in the plasma membrane for CARs to interact and work effectively. A DEG localization analysis was performed to determine the protein position of each expressed gene. Results of the GO term analysis showed 60 selected plasma membrane-related genes (PMGs), and their confidence scores were analyzed using the GeneCard database. The result was an average confidence score of 4.6, with details of 56 genes at > 3 and 4 genes at 3. This means that PMGs are known to have a high probability of being found in plasma cell membranes (Figure 1F). The oncogenicity of PMGs was analyzed based on their expressions in OC and normal samples. As a result, it was found that 21 genes were highly expressed and 25 genes were poorly expressed in OC. In contrast, 14 expressed genes did not significantly differ in expression levels between OC and normal tissue in the database. (Supplementary Figure 1) To analyze further, 18 CAR clinical trial antigen genes were selected as control genes, which were examined for their associations with 19 highly expressed genes (*CD24* and *FOLR1,* which had been used in clinical trials, so they were categorized as control genes) by gene ontology (GO) terms, pathways, expressions, and correlations. (Figure 2A, 2B). The control genes listed have been or are being developed as treatment targets using CAR T cells [14–24]. We obtained the predicted genes in this study and compared their potential bioactivity against control genes. (Table 2, Figure 2C).

**Table 2:** Clinical trial antigens for chimeric antigen receptors (CARs) and used as control genes.

**Gene**	**NCT**	**Gene transfer**	**Reference**
*CEACAM5*	01212887	RV	[14]
*CXCR1*	n.p	TF	[15, 16]
*EGFR*	01869166	LV	[14]
*FSHR*	n.p	RV	[16]
*KLRK1*	03018405	n.p.	[14]
*PDL1*	n.p	LI	[16, 17]
*ANXA2*	n.p	LV	[16, 18]
*CD133*	02541370	RV	[14]
*CTAG1B*	02366546	RV	[14]
*ERBB2*	01935843	LV	[29–31]
*L1CAM*	n.p	LV	[16, 20]
*MAGEA4*	02096614	RV	[14]
*MSLN*	02580747	RV, mRNA, LV	[14, 16, 21]
*MUC16*	02498912	RV, LI	[14, 16, 17]
*TPBG*	n.p	LV	[16, 22]
*WT1*	00562640	n.p.	[14]
*CD24*	n.p	LV	[14, 23]
*FOLR1*	00019136	RV, LV	[14, 16, 24]

Abbreviation: RV, retroviral vector; LV, lentiviral vector; GT, mRNA gene transduction; TF, mRNA transfection; LI, lentiviral infection; n.p., not provided.

### Enrichment analysis of predicted and control genes

Bioactivity, diseases, and expression-specific analyses were carried out to analyze the potential of these 37 genes (highly expressed genes are now called predicted genes). Based on a bioactivity enrichment and pathway analysis, control genes were predicted to have GO enrichment, including T cell costimulation (GO: 0031295) and renal filtration cell differentiation (GO: 0061318); the predicted pathways were endocytosis (hsa04144), GPCR downstream signaling (R-HAS-388396), and extracellular vesicle-mediating signaling in recipient cells (WP2870). Meanwhile, predicted genes were predicted to have GO enrichment, including endocytosis (GO:0006897), inorganic anion transport (GO:0015698), myelination (GO:0042552), metal ion transport (GO:0030001), and mitotic cell cycle process (GO:1903047). Also, the predicted genes induced a pathway in the epithelial-to-mesenchymal transition in colorectal cancer (WP4239). Furthermore, based on the top evidence of GO BP, the predicted gene was predicted to induce fewer BPs than the control gene. According to these results, it could also be predicted that the BP data related to the predicted gene were still limited, so the chance of discovery was higher (Figure 2E, 2F).

Additionally, based on a disease-related gene analysis result, it was found that the control gene was related to the top 20 diseases by confidence scores, compared to the predicted gene, which was only predicted to be related to nine diseases. Based on the confidence scores, C1368683: epithelioma, C0025500: mesothelioma, and C0007138: carcinoma, was the disease with the highest confidence score, where genes in both groups were predicted to play roles in the disease. In addition, the control gene was only expressed (*p* < 0.05) in lung (PGB:00018). In contrast, the predicted gene was expressed (*p* < 0.05) in renal epithelial (PGB:00119), colon tissue (PGB:00007), and K562 cell line [25], which are lymphoblast cells isolated from the bone marrow of a 53-year-old chronic myelogenous leukemia patient (Figure 2G, 2H).

### Protein-protein interaction (PPI) network analysis

**Figure 3:**
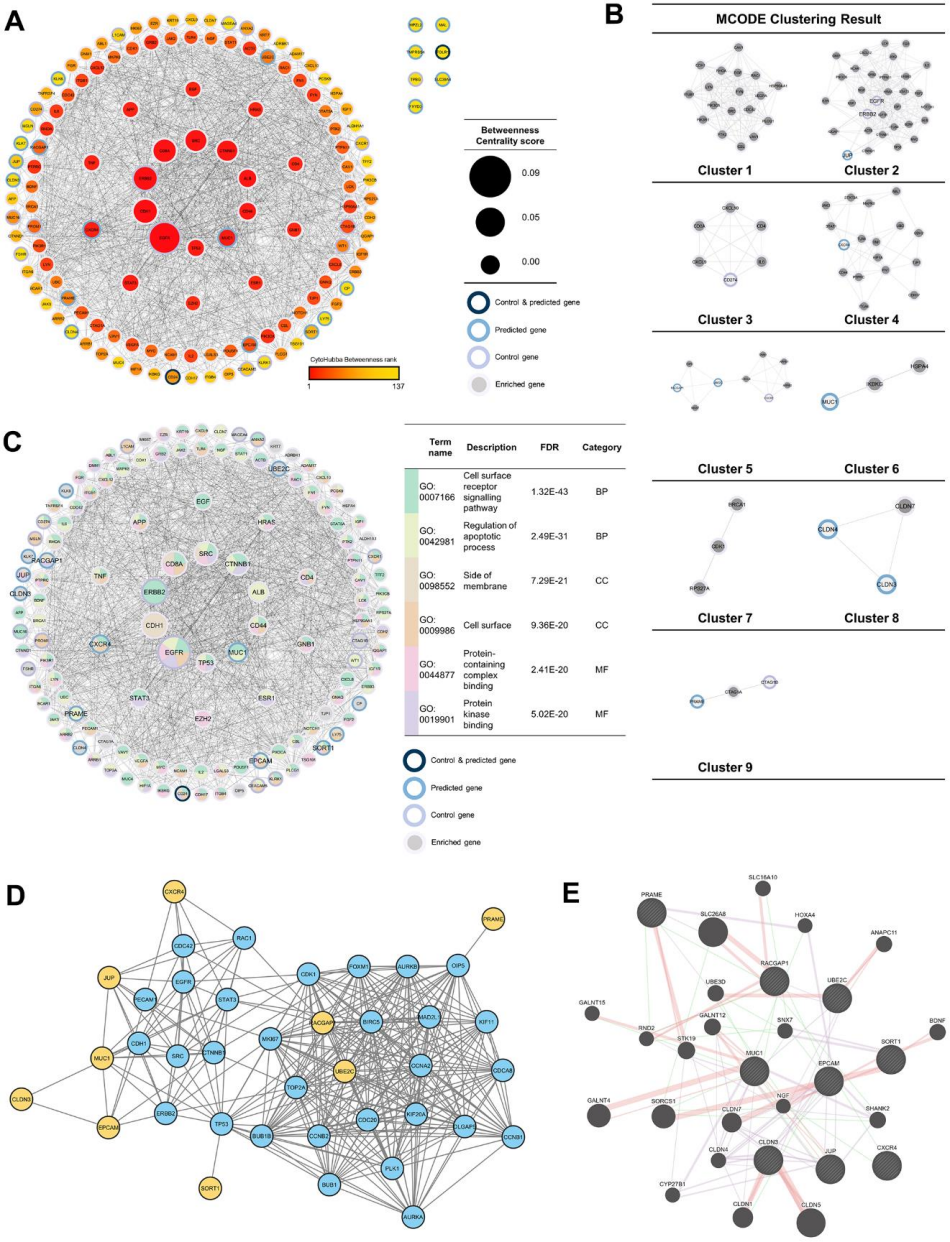
**Protein-protein interaction (PPI) network analysis.** (**A**) PPI network of 37 enriched genes (20 control and 17 predicted genes) with 100 genes, which were analyzed with NetworkAnalyzer and CytoHubba for node and edge scoring. Nine genes with the highest betweenness-centrality scores were designated as TAAr. (**B**) Clustered network with the Molecular Complex Detection (MCODE) algorithm. (**C**) Gene ontology (GO) terms related to control and predicted genes. (**C**) GO term related to control and predicted genes. (**D**) Interaction network of the TAAr gene: yellow genes are TAAr, and blue genes are interactor proteins. (STRING database, high confidence: 0.7). (**E**) GeneMania network of TAAr. The nine biggest nodes with shading are TAAr genes. Edge width is labeled for confidence scores.

To discover more pathways that the 37 genes may induce, a network was built by adding 100 neighboring genes as enriched genes. The PPI network analysis was used because it was proven to be a valuable tool in enhancing the efficacy of CAR T cell therapy by providing insights into critical protein interactions [26], optimizing T cell functions [27], and identifying potential therapeutic targets [28]. Based on the network formed, it was found that *EGFR* and *ERBB2* were genes with the highest betweenness-centrality scores in the control group, while *MUC1* and *CXCR4* were genes with the highest betweenness-centrality scores among predicted genes. In addition to those two genes, *EPCAM, RACGAP1, UBE2C, PRAME, SORT1, JUP*, and *CLDN3* sequentially had the top seven betweenness-centrality scores. Based on MCODE clustering, it was found that there were nine clusters in the main network. Some of the predicted genes in the cluster included *JUP* (cluster 2), *CXCR4* (cluster 4), *RSCGAP1* and *UBE2C* (cluster 5), *MUC1* (cluster 6), *CLDN3*, and *CLDN4* (cluster 8) (Figure 3A, 3B). Furthermore, GO and pathway analyses were also carried out to evaluate which bioactivities and pathways were associated with this network. Based on the GO analysis, the top two from each GO category are shown in Figure 3C. At the same time, *MUC1, CXCR4, EPCAM, RACGAP1, UBE2C, PRAME, SORT1, JUP*, and *CLDN3* were labeled as the recommendation of tumor-associated antigens (TAAr) in a subsequent analysis.

**Figure 4:**
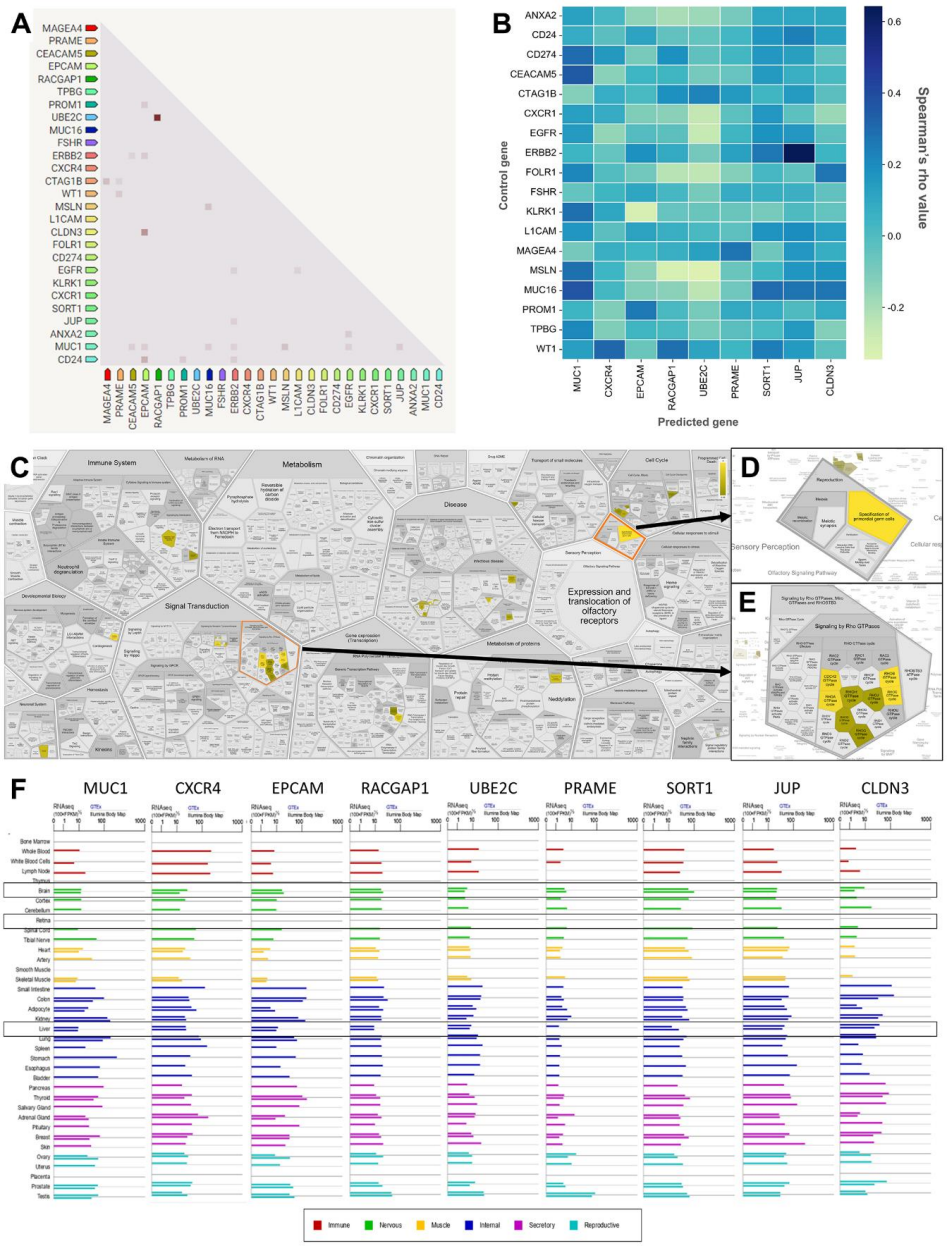
**Correlation, co-expression, and pathway analysis of TAAr.** (**A**) Co-expressed genes are colored by a dot; a darker dot means a higher co-expression score. Co-expression score: the higher the score, the higher the probability that co-expression will occur. (**B**) Spearman’s correlation scores for gene expressions in ovarian cancer (OC), which are comprised of 27 proteins consisting of nine TAAr and 18 control genes. (**C**) The complete pathway of the Reactome database related to TAAr. This pathway was constructed with the Voronoi tessellation method termed ReacFoam, which provides user-friendly access and visualization. (**D**) Pathway related to reproduction where the specification of primordial germ cells pathway is located. (**E**) Rho GTPase-related and neighbor pathways. (**F**) The expression data of TAAr in brain, retina, and liver tissue, provided by GeneCard, were used to analyze the possible toxic effects of targeting TAAr.

Furthermore, to further explore the TAAr genes, co-expression and correlation analyses were carried out on control genes; this analysis was carried out to see relationships between gene expressions that occurred. Based on a STRING co-expression analysis in *Homo sapiens* (cutoff, high confidence: 0.7), it was found that several TAAr genes were co-expressed with control genes. The top five co-expression scores were for *RACGAP1-UBE2C, CLDN3-EPCAM, CD24-EPCAM, CTAG1B-MAGEA4*, and *MSLN-MUC1*. Then, a correlation analysis was performed to explore correlations of TAAr with control genes specific to the OC sample. TAAr interactions with control genes with the highest positive correlation scores were *JUP-ERBB2, MUC1-MUC16, MUC1-CEACAM5, CXCR4-WY1*, and *SORT1-MUC16*. In contrast, interactions with the lowest negative correlation scores were *UBE2C-MSLN, EPCAM-KLRK1, RACGAP1-MSLN, UBE2C-EGFR*, and *UBE2C-CXCR1* (Figure 4A, 4B).

A further co-expression analysis was conducted using GeneMania to investigate other genes correlated with TAAr expressions. We obtained other proteins, CLDN1, CLDN4, CLDN7, cytochrome P450 family 27 subfamily B member 1 (CYP27B1), polypeptide N-acetylgalactosaminyltransferase 12 (GALNT12), Homeobox A4 (HOXA4), nerve growth factor (NGF), Rho family GTPase 2 (RND2), SSH3 and multiple ankyrin repeat domain 2 (HANK2), solute carrier family 26 member 8 (SLC26A8), sorting nexin 7 (SNX7), serine/threonine-protein kinase 19 (STK19), and ubiquitin protein ligase E3D (UBE3D), which developed interactions with and were co-expressed with TAAr. Several other proteins appeared in the pathway we highlighted for each TAAr, but others did not, so we will not examine them further (Figure 3D, 3E and Table 3).

**Table 3:** Networks according to a GeneMania analysis.

**Network**	**Interaction from**	**Coverage**	**Proteins**
Co-expression	Burington-Shaughxnessy-2008	25.46%	CLDN3, CYP27B1, EPCAM, HOXA4, JUP, MUC1, PRAME, SHANK2, SNX7, SORT1, UBE2C
Co-expression	Dobbin-Giordano-2005	14.07%	CLDN4, CLDN7, EPCAM, JUP, MUC1, SNX7, SORT1
Co-expression	Bild-Nevins-2006 B	11.68%	CLDN3, CLDN4, JUP, MUC1, STK19
Co-expression	Ramaswamy-Golub-2001	10.98%	CLDN3, CLDN4, EPCAM
Co-expression	Perou-Botstein-2000	8.30%	CLDN1, CLDN3, CLDN4, CLDN7, EPCAM, MUC1
Co-expression	Wu-Garvey-2007	8.16%	CLDN3, CLDN4, CLDN7, CYP27B1, MUC1, NGF, STK19, UBE2C
Co-expression	Roth-Zlotnik-2006	7.68%	CLDN3, CLDN4, CLDN7, EPCAM, GALNT12, PRAME, SLC26A8
Co-expression	Wang-Cheung-2015	2.33%	CLDN4, CLDN7, JUP, NGF, RACGAP1, SHANK2, UBE2C
Co-expression	Arijs-Rutgeers-2009	0.49%	CLDN1, CLDN3, CLDN4, GALNT12, MUC1, PRAME, RACGAP1, RND2, UBE2C, UBE3D
Physical Interaction	Biogrid-small-scale-studies	4.13%	ANAPC11, BDNF, CLDN1, CLDN3, CLDN5, GALNT4, GALNT15, JUP, MUC1, NGF, PRAME, RACGAP1, RND2, SLC26A8, STK19, SORCS1, SORT1, UBE2C, UBE3D
Physical Interaction	IREF-hprd	2.14%	ANAPC11, BDNF, CDLN1, CLDN3, CLDN5, CLDN7, EPCAM, GALNT12, GALNT15, JUP, NGF, PRAME, RACGAP1, RND2, SLC16A10, SLC26A8, SORT1, STK19, UBE2C
Physical Interaction	IREF-biogrid	1.79%	ANAPC11, CLDN1, CLDN3, CLDN5, GALNT4, GALNT12, MUC1, JUP, RACGAP1, RND2, UBE2C
Genetic Interaction	Lin-Smith-2010	2.80%	CLDN1, CXCR4, EPCAM, GALNT13, GALNT15, HOXA4, JUP, MUC1, NGF, PRAME, RACGAP1, RND2, SHANK2, SLC16A10, SLC26A8, SNX7, SORCS1, UBE3D

*^)^ Underlined proteins are the recommendation of tumor-associated antigens (TAAr).

### Enrichment analysis of a novel tumor-associated antigen candidate for ovarian cancer

Three pathways with the highest confidence scores based on Reactome were primordial germ cell specification, RhoB GTPase cycle, and RhoC GTPase cycle, with TAAr genes involved in these pathways being *CXCR4, JUP*, and *RACGAP1*. Complete information is given in Table 4. Primordial germ cell specifications are developmental processes of primordial germ cells (PGCs) that occur around the third to fourth week of pregnancy in humans to become gametes. In the process of becoming gametes, PGCs undergo various metabolic cycles in which the expression or suppression of each gene has an important role, and disruption of this critical role can cause developmental disorders, from disabilities to an inability to fertilize and germ cell-specific tumors [33, 34]. Furthermore, the Rho GTPase cycle plays a role in various cellular responses, including regulating cell migration. This cycle is controlled by Rho family GTPases, members of the Ras superfamily, which can be grouped into two typical and atypical subdivisions. Rho family members in the typical subdivision are RhoA, Cdc42, and Rac1, with a cycle related to GTP-bound activation and GDP-bound inactivation conformation. Next, Rho family members in the atypical subdivision are those proteins that possess amino acid substitutions that affect their ability to interact with GTP or GDP and are regulated by certain mechanisms [35]. Also, the Rho GTPase cycle is known to play an essential role in regulating the actin cytoskeleton, where the actin cytoskeleton plays a role in cellular processes such as cell division, migration, chemotaxis, and endocytosis [36, 37]. Finally, the link of this pathway to OC is through the process of tumor cell growth, where tumor germ cells can end up as ovarian germ cell tumors due to unregulated metabolic processes, and Rho GTPases can increase cellular activity related to cell division, proliferation, and migration [37, 38].

**Table 4:** The most significant pathways of the Reactome.

**Pathway name**	**Entities found^a)^**	***p*-value**	**FDR**
Specification of primordial germ cells	2 / 64	< 0.001	0.175
RhoB GTPase cycle [32]	2 / 75	0.001	0.175
RhoC GTPase cycle [32]	2 / 85	0.002	0.175
Formation of definitive endoderm	3 / 85	0.002	0.175
RhoA GTPase cycle [32]	2 / 154	0.005	0.193

^a)^Entities found are a count of entities input with pathway-related entities in Reactome. FDR, false discovery rate.

**Figure 5:**
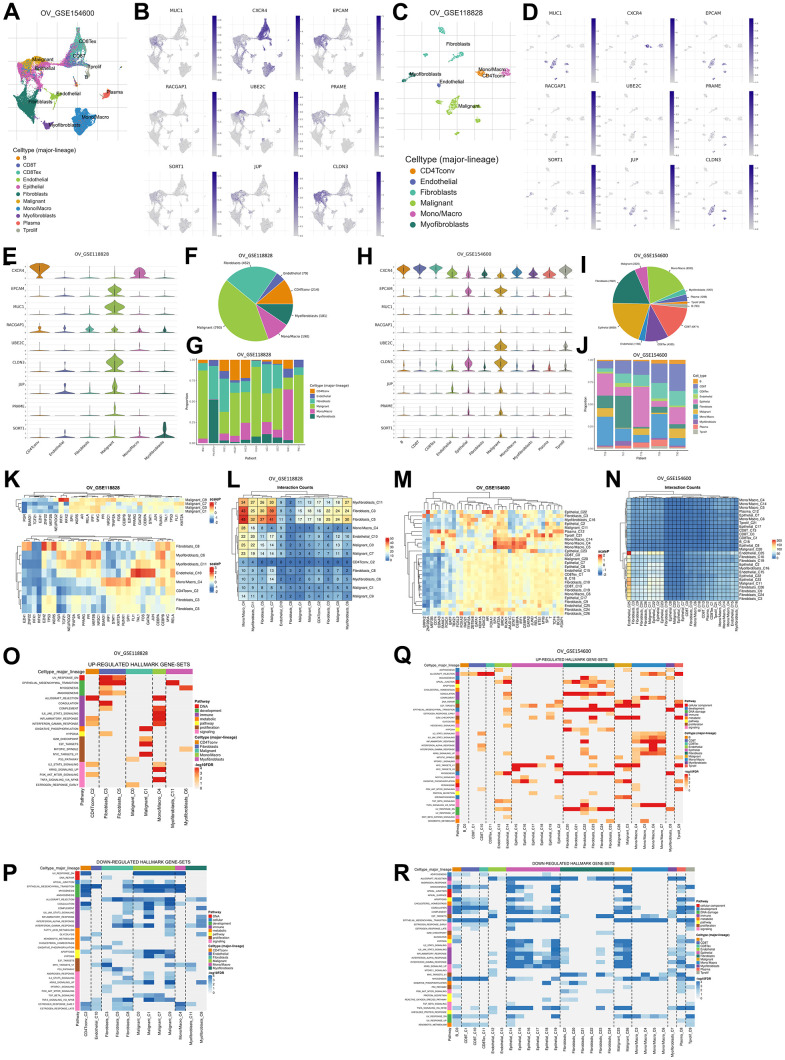
**Gene expression levels are based on single-cell RNA sequencing based on a human sample based on the TISCH2 database.** (**A**) Sample from GSE154600 based on 5 non-treatment patients, 42583 cells, analyzed with the 10x genomics platform. (**B**) UMAP plot of nine TAAr on GSE154600, where EPCAM, MUC1, RACGAP1, CLDN3, and JUP are highly expressed in malignant cell samples. (**C**) Sample from GSE118828 based on 9 non-treatment patients, 1909 cells, analyzed with the Smart-Seq2 platform. (**D**) UMAP plot of nine TAAr on GSE118828, where CXCR4, MUC1, EPCAM, UBE2C, CLDN3, JUP, PRAME, and SORT1 are highly expressed in malignant cell samples. (**E**) Violin diagram of TAAr expression in each sample cell in GSE118828. (**F**, **G**) Diversification and comparison of the number of samples analyzed in GSE118828. (**H**) Violin diagram of TAAr expression in each sample cell on GSE154600. (**I**, **J**) Diversification and comparison of the number of samples analyzed on GSE154600. (**K**) Transcription factor induced by TAAr in GSE118828: the higher the induction, the redder it is. (**L**) Number of cell-cell interactions in sample GSE118828. (**M**) Transcription factor induced by TAAr in GSE154600: the higher the induction, the redder it is. (**N**) Number of cell-cell interactions in the GSE154600 sample. (**O**, **P**) The genetical hallmarks that are up-downregulated based on the TAAr gene in GSE118828. (**Q**, **R**) The genetical hallmarks that are up-downregulated based on the TAAr gene in GSE154600.

Also, based on an ingenuity pathway analysis (IPA), we obtained 15 pathways with the highest confidence scores out of three annotations (Table 5). IPA results showed predicted pathways related to the nine recommended antigens, especially toxicity pathways that may occur with or be associated with these proteins. Based on toxicity results, it was observed that the liver and kidneys may be organs that need to be examined for adverse effects that may occur. To verify expressions of TAAr in brain, retina, and liver tissues, we obtained data from GeneCard (Figure 4F). These data showed that the TAAr had the lowest RNAseq counts in the retina, while the brain and liver had varied RNAseq counts (100× FPKB). We highlight three proteins, SORT1, JUP, and CLDN3, with upper-middle RNAseq expressions in these two organs. CAR-T-cell therapy may have adverse effects on these three organs. Neurotoxicity is a frequent complication after CAR-T-cell therapy, and the exact cause of which is still under research and debate. Apart from that, the neurotoxicity of CAR-T-cell therapy is probably due to high cytokine levels in the brain and cerebrospinal fluid associated with the blood-brain barrier [39]. Also, CAR-T-cell therapy commonly induces neurological complications termed immune effector cell-associated neurotoxicity syndrome (ICANS). The ICANS is a significant concern in the context of chimeric antigen receptor (CAR) T cell therapy [40]. Liver toxicity was also reported in an earlier CAR-T-cell therapy clinical trial. Three metastatic renal carcinoma patients who received autologous T cells transduced with CAR-targeting carboxyanhydrase-IX (CAIX) experienced cholangitis due to T-cell infiltration around the bile ducts, because bile duct epithelial cells unusually express CAIX [41, 42]. Furthermore, CAR-T-cell therapy was also reported to affect the patient, even though it does not seem to be severe. Treatment results in 1421 cases showed that 28 of them had eye disorders, with abnormalities in the form of vision impairment or changes, impaired pupil responses, papilledema, mydriasis, photophobia, and visual tracking test abnormalities [43, 44]. Then, the final enrichment results, namely single-cell RNA sequencing (scRNA-seq) analysis of the tumor microenvironment (TME). Single-cell RNA analysis provides numerous advantages over traditional bulk RNA sequencing methods. This technology has been pivotal in detecting novel cell types with distinct expression signatures and understanding the stochasticity of gene expression within a cell population. Additionally, single-cell RNA sequencing allows for differential expression analysis, clustering, cell type annotation, and pseudotime analysis at a single-cell level, leading to significant progress in the field of transcriptomics [45, 46]. According to this analysis, the result indicated that EPCAM, MUC1, UBE2C, and CLDN3 were highly expressed in malignant samples in two scRNA-seq databases, thereby increasing the potential of TAAr (Figure 5).

**Table 5:** The five canonical, disease and function, and toxicity pathways based on an ingenuity pathway analysis result of nine genes.

**Annotation**	**Name**	***p*-value**
Canonical pathway	Granulocyte adhesion and diapedesis	2.34E-03
Canonical pathway	Leukocyte extravasation signaling	2.75E-03
Canonical pathway	Agranulocyte adhesion and diapedesis	2.87E-03
Canonical pathway	Sertoli cell-Sertoli cell junction signaling	3.14E-03
Canonical pathway	FAT10 cancer signaling pathway	2.11E-02
Disease and function	Primary ovarian cancer	1.17E-10
Disease and function	Serous ovarian adenocarcinoma	1.77E-09
Disease and function	Primary clear cell ovarian carcinoma	3E-09
Disease and function	Primary serous ovarian carcinoma	3.63E-08
Disease and function	Clear-cell ovarian carcinoma	2.92E-07
Toxicity	Hepatic fibrosis	8.64E-03
Toxicity	Hypoxia-inducible factor signaling	2.90E-02
Toxicity	Increases liver damage	4.18E-02
Toxicity	Increases renal damage	4.63E-02
Toxicity	Decreases transmembrane potential of mitochondria and the mitochondrial membrane potential	6.10E-02

## DISCUSSION

CAR T cell therapy has shown promise in treating hematological malignancies, with the potential for sustained remissions and improved clinical outcomes [47]. This is due to the healing effectiveness of CAR treatment in acute lymphocytic leukemia patients, which can reach 92%. CARs work by targeting the tumor associated antigen (TAA) found on the cell surface and bypassing the recognition process with MHC class I or II [48]. However, the development of and research into CARs in solid tumors are still limited, including in OC patients. OC is a type of cancer and an orphan drug disease with a high mortality rate due to malignancies [49, 50].

Thus, researching target genes for therapy (biomarkers) remains essential to optimize treatment efficacy and costs [51]. Based on our bioinformatic analysis, we found nine novel TAAr genes, including MUC1, CXCR4, EPCAM, RACGAP1, UBE2C, PRAME, SORT1, JUP, and CLDN3, that have been identified and may have the potential to be further studied for their role in OC clinically to help optimize OC therapy.

The first of the nine TAAr is Claudin 3. Regulation of claudin 3 protein (CLDN3) (gene: *CLDN3*) in ovarian cancer cells is a complex process influenced by genetic and epigenetic factors. CLDN3, a tight junction protein, plays an important role in cellular interaction and migration. Studies have shown that this gene is associated with the epithelial-to-mesenchymal transition (EMT), a process that induces cell migration or the aggressiveness of cancer cells [52]. Research has shown that the transcription factor Sp1 plays a crucial role in controlling the expression of CLDN3. Moreover, epigenetic modifications, such as DNA methylation and histone alterations, are significant in regulating the CLDN3 promoter in ovarian cancer cells [53, 54]. This complex regulatory landscape results in CLDN3’s overexpression in epithelial ovarian cancer (EOC), and ovarian epithelial inclusion cysts may potentially serve as a marker for malignancy but also as a potential target for immunotherapy [55]. The aberrant expression patterns and the pivotal role of CLDN3 in maintaining cellular polarity and ion flux underscore its significance in cancer pathology and as a candidate for targeted CAR-T cell therapy, aimed at improving treatment specificity and efficacy against ovarian cancer. Integrating these molecular insights with the broader application of CAR-T therapy underscores the potential for targeting CLDN3 in ovarian cancer treatment. The overexpression of CLDN3 in ovarian cancer, contrasted with its low levels in normal tissues and benign conditions, positions it as a distinct antigen for CAR-T cell targeting. This approach could lead to the development of more precise and effective therapeutic strategies, leveraging the unique expression profile of CLDN3 to minimize off-target effects and enhance the therapeutic window. The ongoing research into the regulation and function of CLDN3 in ovarian cancer not only deepens our understanding of the disease’s molecular underpinnings but also paves the way for novel, targeted immunotherapies that could significantly impact patient outcomes in ovarian cancer, a malignancy notorious for its resistance to conventional treatments [53, 56, 57].

Second, membrane protein that could potentially become an ovarian cancer biomarker is CXCR4, which can activate several downstream proteins. The C-X-C Motif Chemokine Receptor 4 (CXCR4) (gene: *CXCR4*) plays various biological roles, specifically in cell migration, hematopoiesis, cell homing, retention in the bone marrow, and cell homing. It also directly controls cell proliferation of non-hematopoietic cells [58]. Studies have demonstrated that a substantial majority of advanced EOC patients exhibit expression of CXCR4 and CXCR7, with over half showing expression of the full CXCR4-CXCL12-CXCR7 axis [59]. This expression pattern is closely associated with poor progression-free and overall survival rates, particularly in EOC patients [60, 61]. Focusing on CXCR4, this gene is linked to promoting ovarian cancer aggressiveness. Several studies report that inhibiting CXCR4 by its antagonist reduces tumor growth *in vitro* and *in vivo* by hindering cell proliferation, migration, and invasion [62]. Such findings underscore the critical role of CXCR4 in tumor progression and suggest its components as promising targets for novel therapeutic strategies, including CAR-T therapy.

The third TAAr is epithelial cell adhesion molecule protein (EpCAM) (gene: *EPCAM*). EPCAM is a membrane protein considered in the past to play a role in regulating cellular communication as an adhesion molecule, but now this protein has been shown to have various biological functions, including in the regulation of cell proliferation and cancer stemness [63]. The elucidation of EpCAM’s role in ovarian cancer presents a transformative insight into the mechanisms of tumor aggression and chemoresistance. EpCAM^+^CD45^+^ cells, identified in the ascitic fluid of patients with serous EOC, not only manifest a drug-resistant phenotype but also display invasive characteristics through the overexpression of genes like SIRT1, ABCA1, and BCL2. This phenotype’s ability to evade immune surveillance by overexpressing major histocompatibility complex class I antigen accentuates its potential as a pivotal player in ovarian cancers pathogenesis and persistence. The complex interplay between these cells and the tumor microenvironment, especially the influence of non-tumor cell exosomes in fostering drug resistance and invasiveness, underscores the necessity of targeted therapeutic strategies aimed at this phenotype [64]. Related to their expression, EpCAM is overexpressed in comparison between 30 EOC and 15 normal ovary tissues based on immunohistochemistry evaluation. Then, according to those studies indicating its role in cancer progression, metastasis, and drug resistance [65]. The RT-PCR primer for EpCAM, MUC1, and PRAME can be seen in Supplementary Table 2 [66].

The association of EpCAM with pathways like PI3K/AKT signaling and its regulation by factors such as EGF via ERK1/2 signaling elucidates its part in tumor progression and resistance mechanisms. Moreover, EpCAM’s involvement in tumor immune modulation, particularly its ability to resist natural killer cell-mediated cytotoxicity, highlights its significance in tumor immune evasion strategies. The exploration of EpCAM-targeted therapies, such as the bispecific T-cell engaging antibodies (BiTE) that show efficacy against ovarian cancer cells, underscores the potential of EpCAM as a target for innovative therapeutic interventions. These findings collectively emphasize the necessity for continued research into EpCAM-focused therapies, aiming at disrupting the intricate mechanisms through which EpCAM+ cells contribute to ovarian cancer’s malignancy and chemoresistance, thereby opening avenues for more effective and personalized treatment modalities for EOC [64, 67, 68]. By focusing on EpCAM expression and their molecular pathways, there emerges an opportunity to enhance the efficacy of treatments and improve patient outcomes, pointing towards a targeted approach to managing EOC. Also, integrating the knowledge from the study on EpCAM’s impact on chemotherapy response and clinical outcomes with findings from previous research sheds light on a broader spectrum of EpCAM’s roles in EOC’s molecular landscape.

The fourth TAAr is Junction Plakoglobin, also known as plakoglobin (JUP) (gene: *JUP*). JUP is a part of the adhesion molecule that can affect the Ras, Hedgehog, and Wnt signaling pathways, although the activity remains unclear yet [69]. JUP emphasizing its potential as a specific early detection biomarker for ovarian cancer, uniquely elevated in the ovarian venous blood of patients with early-stage epithelial ovarian carcinomas, particularly serous stage IA+B through III. JUP, part of the Armadillo protein family, crucial for cell adhesion and architecture, shows specificity by not being elevated in early-stage breast cancer. It also interacts with the p53 protein, possibly influencing tumor suppression in ovarian cancer. This suggests JUP could, alongside traditional markers like CA125, improve diagnostic precision, facilitating earlier, more effective treatments. Its role in oncogenic pathways, particularly in cell-cell adhesion and migration, highlights its importance in understanding ovarian cancer’s malignancy and metastasis processes, suggesting a significant potential in refining therapeutic strategies and advancing the efficacy of interventions in ovarian cancer treatment [70].

The next TAAr is the transmembrane glycoprotein mucin 1 (MUC1), (gene: *M*UC1). MUC1 is a mucin family that the body can use it as a lubricant, moisturizer, and physical barrier. MUC1 serves a pivotal role in EOC, significantly influencing tumor metastasis and progression [71]. Characteristically overexpressed in EOC, MUC1 is primarily found on the surface of tumor cells and is underglycosylated, exposing epitopes concealed in non-malignant cells. This unique expression not only positions MUC1 as a critical diagnostic biomarker but also as a promising therapeutic target. The protein interacts with various oncogenic signaling pathways enhancing cell survival, proliferation, and metastasis. Such interactions suggest that MUC1 could enhance the specificity and efficacy of CAR-T cell therapy by targeting these aberrant [72, 73].

Another TAAr is the Preferentially Expressed Antigen in Melanoma protein (PRAME, gene: *PRAME*). PRAME has emerged as a significant tumor-specific antigen for ovarian cancer therapy, particularly in high-grade serous carcinoma (HGSC). Identified for its high expression relative to healthy tissues, PRAME’s promoter hypomethylation correlates with increased mRNA levels across various stages and grades of ovarian cancer. This epigenetic regulation suggests a pivotal role for PRAME as both a therapeutic target and a prognostic marker, potentially enhancing survival outcomes in HGSC patients. The specific targeting of PRAME through T-cell receptor (TCR) therapies, leveraging allogeneic HLA T-cell repertoires, has demonstrated potent anti-tumor reactivity *in vitro* and *in vivo*, underscoring the antigen’s viability as a focus for advanced immunotherapeutic strategies. The dual focus on PRAME and another promising target, CTCFL, reveals the need for precision in the balance of specificity and efficacy in TCR therapies for ovarian cancer. The selective recognition and destruction of ovarian cancer cells by CTCFL-specific TCRs without impacting healthy cells further exemplify the potential of such targeted therapies. These findings prompt continued research into the mechanisms regulating PRAME expression and its functional impact on the disease, offering pathways to enhance the efficacy of PRAME-directed treatments. Despite its frequent expression at both mRNA and protein levels, the lack of correlation between PRAME protein expression and clinical outcomes in ovarian cancer highlights the complexity of its role, emphasizing the necessity for further studies to optimize therapeutic approaches and validate PRAME as a key molecule in targeted ovarian cancer therapy [74, 75].

The seventh TAAr is the Rac GTPase activating protein 1 (RACGAP1, gene: *RACGAP1*). RACGAP1 plays a critical role in the progression and prognosis of epithelial ovarian cancer (EOC), predominantly expressed in the nuclei of tumor cells and associated with advanced tumor stages, high pathological grades, and lymph node metastasis. The overexpression of RACGAP1 correlates with shortened overall survival and increased disease recurrence. Functionally, RACGAP1 enhances the activation of RhoA and Erk proteins, promoting the migratory and invasive capabilities of EOC cells, which underscores its potential as a therapeutic target and a biomarker for disease progression. This study establishes RACGAP1 as a novel, potent target for therapeutic intervention and necessitates further experimental and clinical evaluations to explore RACGAP1 inhibitors or modulators to improve ovarian cancer treatment strategies [76].

The next TAAr is Sortilin 1 (SORT1, gene: *SORT1*), a transmembrane protein. SORT1 is highly expressed in various cancers, including ovarian and endometrial tumors. Its role in internalizing ligands, such as the TH19P01 peptide linked to the therapeutic agent docetaxel in the TH1902 compound, is critical. This interaction enhances the delivery of the cytotoxic agent directly to SORT1-expressing cancer cells, improving the efficacy of the treatment while reducing off-target effects and systemic toxicity. Clinical studies and models have demonstrated that TH1902, by leveraging SORT1-mediated endocytosis, notably suppresses tumor growth more effectively than conventional chemotherapy agents like docetaxel alone, suggesting its potential as a targeted therapy for SORT1-positive gynecological cancers. Further research into the ectopic expression of SORT1 in ovarian carcinoma has shown a four-fold increase in its gene expression in carcinoma tissues compared to non-malignant tissues, highlighting its absence in normal ovarian tissue and its potential as a novel tumor marker. The predominant cell surface localization of SORT1, as opposed to the expected ER-Golgi compartment, suggests a significant role in tumor cell interaction with the microenvironment, potentially affecting tumor growth and metastasis. These findings underline the importance of SORT1 in cancer cell proliferation pathways and the need for targeted therapeutic approaches that exploit these unique properties of SORT1, offering a promising avenue for refining diagnostic tools and therapeutic strategies in the management of ovarian carcinoma [77, 78].

The last TAAr is the ubiquitin-conjugating enzyme E2C (UBE2C, gene: *UBE2C*), which emerges as a pivotal oncogene in ovarian cancer, significantly influencing tumor malignancy and resistance to cisplatin chemotherapy by interacting with Cyclin-Dependent Kinase 1 (CDK1). Overexpression of UBE2C in ovarian cancer correlates with adverse clinical outcomes, including elevated tumor grades and diminished survival rates. The silencing of UBE2C in ovarian cancer cell lines markedly reduces cell proliferation and augments apoptosis, primarily by inducing G2/M phase arrest and reducing CDK1 expression. This underpins the functional linkage between UBE2C and CDK1, substantiated by their correlated expression in tumor tissues. Hence, targeting UBE2C may substantially bolster the efficacy of cisplatin treatments, reversing drug resistance and curbing tumor growth. These insights significantly contribute to the expansion of CAR T-cell therapy for ovarian cancer by pinpointing UBE2C and its associated molecular pathways as promising therapeutic targets, potentially enhancing the precision and effectiveness of these innovative treatments in managing a malignancy notorious for its grim prognosis and limited therapeutic options [79]. The more detailed descriptions of TAAr findings and studies can be seen in Supplementary Table 1.

TAAr might have a role as a tumor-specific antigen that is modulated by the patient’s genetic background, the TME, and a multitude of other elements. This complexity serves as a significant barrier to the development of targeted therapies for OC treatment [80]. However, overexpression of these proteins, especially EPCAM, MUC1, UBE2C, and CLDN3, shows potential as promising biomarkers for early detection of disease or assessment of therapeutic response. Although, all of nine target proteins demonstrated their roles in oncogenic pathways and their suitability for development as TAA in CAR treatment. These proteins play distinct roles in biological pathways, but their cumulative dysregulation also might generate the OC malignancy. Formulating a comprehensive molecular target involving the roles and interactions of MUC1, CXCR4, EPCAM, RACGAP1, UBE2C, PRAME, SORT1, JUP, and CLDN3 in the scope of OC initiation and progression was a challenging attempt, because the actual bioactivity is markedly more complicated due to interactions between these and other signaling pathways, cellular heterogeneity within OC (Currently, it is known that there are five categories of ovarian carcinoma, including high-grade serous ovarian carcinoma (HGSOC), endometrioid ovarian carcinoma (EOVC), ovarian clear cell carcinoma (OCCC), low-grade serous ovarian carcinoma (LGSOC), and mucinous ovarian carcinoma (MOC)) [81], and the dynamic influence of the TME, and studying these limitations was pivotal to understanding the forefront of scientific research within the field of OC.

Although we have suggested TAAr as a novel OC biomarker candidate, there are several important limitations in our research related to bioinformatics methods. First, the study relied exclusively on analyses of existing gene expression datasets (GSE data) obtained from the GEO database, which might not entirely represent the heterogeneity of OC patients. Second, the research solely depended on bioinformatics-based oncogenicity predictions, which can lack the ability to capture the complex, dynamic interactions within TME and its influence on the oncogenic potential of the identified proteins. Lastly, the study provides no experimental validation for the novel antigen candidates (TAAr). As a consequence, we recommend conducting the *in vitro* and *in vivo* studies to confirm the potential biomarker of TAAr and to evaluate their molecular pathway in ovarian cancer. Then, a clinical study can be performed to evaluate their novelty as biomarkers of ovarian cancer and their effectiveness as CAR-T cell target antigens.

## CONCLUSIONS

In this research, the landscape of potential antigenic targets for CAR-T-cell therapies in OC has been significantly broadened. By applying bioinformatics methodologies to analyze DEGs and PPIs, the study has highlighted nine proteins (MUC1, CXCR4, EPCAM, RACGAP1, UBE2C, PRAME, SORT1, JUP, and CLDN3) as pivotal proteins in the oncogenic pathways of OC, thereby marking them as compelling antigen candidates for CAR-T-cell interventions. These proteins, exhibited on the plasma membrane and predicted to be oncogenic, provide a high degree of specificity for potential targeted therapies. Nevertheless, as the baseline analysis was computational, additional experimental and clinical validations are essential to corroborate these initial findings and accurately evaluate the efficacy of these proteins as CAR-T-cell antigens. Thus, this study represents a significant leap in the ongoing research for detailed and vigorous immunotherapies for OC.

## MATERIALS AND METHODS

### Data sources

**Figure 6:**
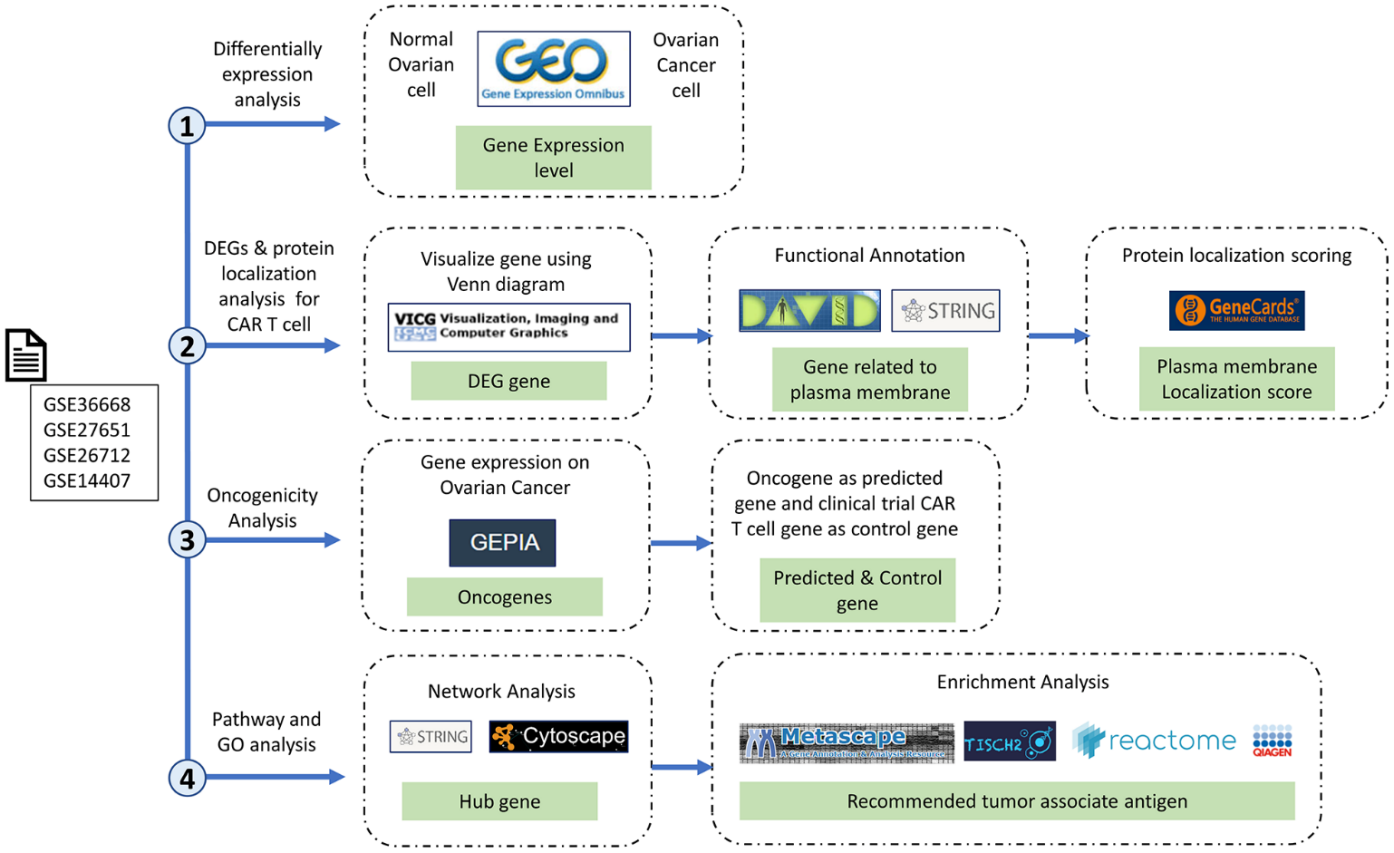
**Research stages and workflow.** The four research red lines are differential expression analysis, differentially expressed genes (DEGs), protein localization analysis, oncogenicity analysis, and pathway and gene ontology analyses. Therefore, in this study, we carried out multilevel screening to reduce the potential for errors or discrepancies later. What we mean by multilevel screening is first looking at the significance of the expression of a gene in normal and cancer samples from the dataset, then checking the significance of the significant gene again in a different database to ensure that the gene is significantly expressed only in cancer cells.

The study was conducted following the general steps detailed in Figure 6. There was a determination of DEGs, followed by examination of protein localization and oncogenicity, and network and enrichment analyses of the recommendation of target genes. The microarray dataset was obtained from the NCBI-GEO database, which is a public gene profile database (https://www.ncbi.nlm.nih.gov/gds/). The four datasets relevant to OC, namely GSE36668, [9] GSE276512, [10] GSE26712, [11, 12] and GSE14407, [13] are datasets related to OC that use three levels on the GPL570 platform: [HG-U133_Plus_2] Affymetrix Human Genome U133 Plus 2.0 Array, and GSE26712 using the GPL96 platform: [HG-U133A] Affymetrix Human Genome U133A Array. The dataset above is used because each dataset is submitted no later than the last 20 years, can be analyzed with the GEO2R tool, and has samples of ovarian cancer tissue and normal cell tissue in one dataset. GSE36668 had 12 samples (eight OC and four normal samples). GSE27651 had 49 samples (43 OC and six normal samples). GSE26712 had 195 samples (185 OC and 10 normal samples). GSE14407 had 24 samples (12 OC and 12 normal samples). In this study, we used a balanced count of data samples between normal and OC samples from each GSE.

### DEG identification

Identification of DEGs was carried out using the GEO2R interactive tool (http://www.ncbi.nlm.nih.gov/geo/geo2r) provided by NCBI to compare a number of sample datasets from the microarray dataset series. The threshold used was logFC > 1.5 and a *p*-value of < 0.05, which were considered to be of statistical significance. Next, GEO2R results were analyzed using Microsoft Excel format to separate genes with the same ID, and online Venn software [82] was used to determine gene intersections of the four datasets, which are DEGs.

### Functional examination of DEGs

Functional annotation analysis of gene ontology (GO) and pathway of DEGs was performed with the Database for Annotation, Visualization, and Integrated Discovery (DAVID; https://david.ncifcrf.gov/). Then, genes associated with the seven GO terms with the highest confidence score (-log10 (FDR)) for GO biological processes (BPs), cellular components (CCs), and molecular functions (MFs) were visualized, and plasma membrane-related genes (PMGs) were selected for analysis. Advanced confidence scores were from the GeneCard database (https://www.genecards.org/). The analysis plays a role in ensuring that the expressed protein is located in the plasma membrane so that it can be targeted as a CAR antigen.

### Oncogenicity prediction analysis

Oncogenicity predictions were carried out on PMGs using Gene Expression Profiling Interactive Analysis (GEPIA2; http://gepia2.cancer-pku.cn/#index). Gene expressions were compared in OC and normal samples. Parameters used were a Log2FC cutoff of 2, a *p*-value cutoff of 0.01, and matching of The Cancer Genome Atlas (TCGA) normal and GTEx data. GEPIA is a web-based tool that provides interactive features such as profile plotting, patient survival analysis, differential expression analysis, similar gene detection, and dimensionality reduction analysis [83]. A highly expressed gene in OC was designated a predicted gene, and predicted genes were used in the next analysis.

### Protein-protein interaction (PPI) network analysis

A PPI network analysis was accomplished using the Search Tool for the Retrieval of Interacting Genes and Proteins (STRING; https://string-db.org/) and Cytoscape 3.10. A network of highly expressed genes in OC was constructed in STRING with an interaction confidence score of > 0.7 [84] and an additional 100 enriched genes. Network PPIs were analyzed with NetworkAnalyzer [85] and CytoHubba [86, 87] to determine the betweenness score of each node. In addition, an MCODE clustering analysis, GO, and pathways were also carried out to more deeply investigate results of enrichment. MCDE clustering was carried out using CluserViz with a degree threshold of 2, a node score threshold of 0.2, a K-core threshold of 2, and a max depth of 100, resulting in nine clusters [88, 89].

### Enrichment analysis

Basic GO and pathway analyses were carried out using the g: Profiler webserver [90]. GO terms and pathways with the best confidence score (FDR) were visualized. A TAAr enrichment analysis was also carried out using Metascape to obtain bioactivity comparisons between control and predicted genes [91, 92], TIMER to acquire co-expression scores of predicted genes to control genes [93], Reactome webserver (https://reactome.org/) and an Ingenuity pathway analysis (IPA) (Qiagen, USA) for analyzing pathway and bioactivity of predicted gene, and an scRNA-seq analysis by Tumor Immune Single-cell Hub 2 (TISCH2) database to provide detailed cell-type annotation at the single-cell level, enabling the exploration of TME across different cancer types focusing on tumor microenvironment (TME) [94], and the SRPlot online tool (https://www.bioinformatics.com.cn/en) to construct and analyze data graphics [95].

### Data availability statement

The data analyzed in this study are freely available and can be accessed in the NCBI database (https://www.ncbi.nlm.nih.gov/). Another set of data presented in this paper is available from the corresponding author upon reasonable request.

## Supplementary Material

Supplementary Figure 1

Supplementary Table 1

Supplementary Table 2
