# Supplementary material for: Discovery of differentially expressed proteins for CAR-T therapy of ovarian cancers with a bioinformatics analysis
Source: Aging (Albany NY). 2024 Jul 18;16(14):11409–33. doi: 10.18632/aging.206024 (PMC11315388; doi:10.18632/aging.206024)
Supplement: Supplementary Figure 1 [file aging-16-206024-s001.pdf]

SUPPLEMENTARY FIGURE

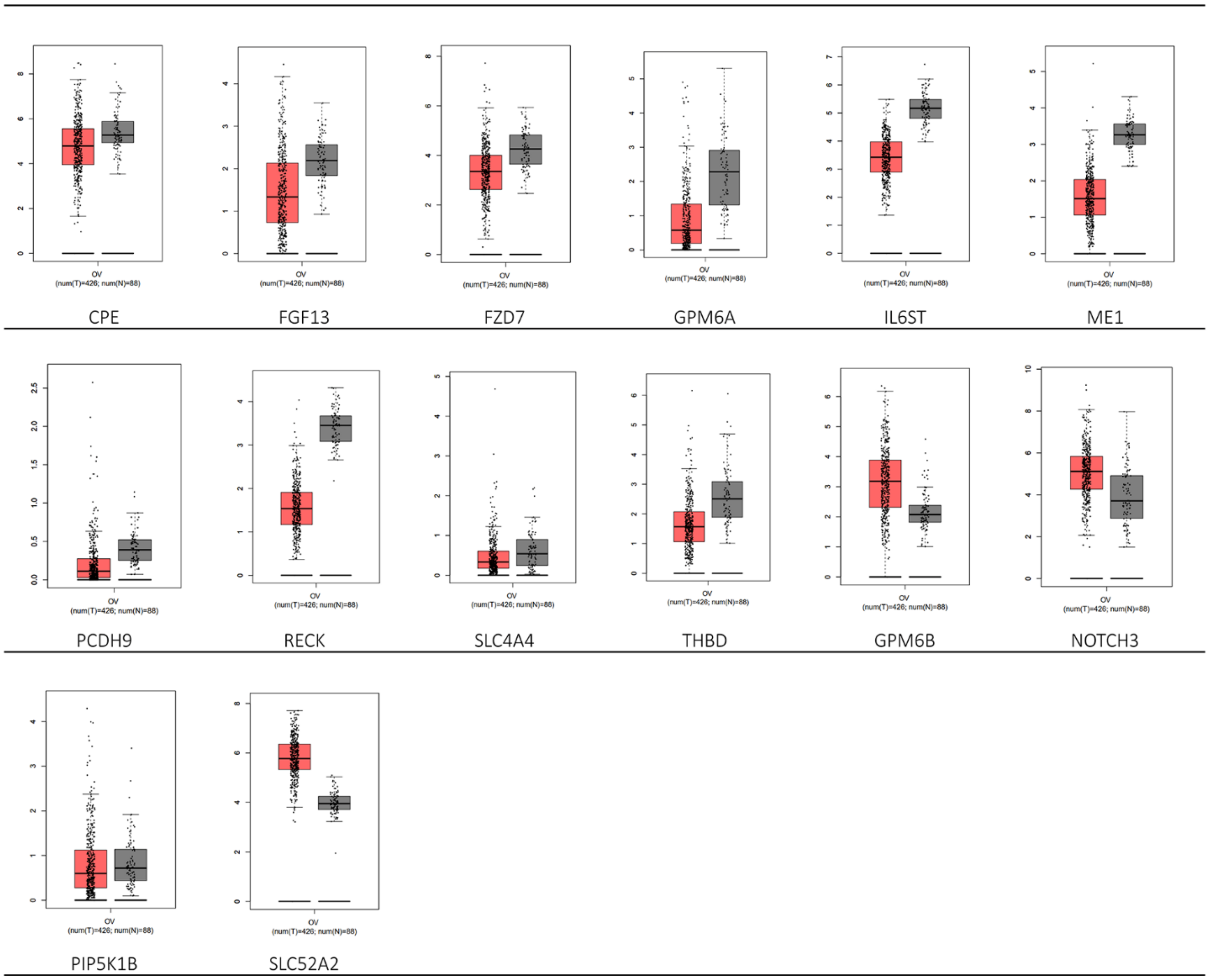

Supplementary Figure 1. Several PMG expressions are not significant based on the ovarian cancer dataset in the GEPIA database.
