# Supplementary material for: Discovery of differentially expressed proteins for CAR-T therapy of ovarian cancers with a bioinformatics analysis
Source: Aging (Albany NY). 2024 Jul 18;16(14):11409–33. doi: 10.18632/aging.206024 (PMC11315388; doi:10.18632/aging.206024)
Supplement: Supplementary Table 1 [file aging-16-206024-s002.docx]

# Supplementary Table 1. The more detailed descriptions of TAAr findings and studies.

| **Gene** | **Research finding** | **Clinical Finding** | **Therapeutic potential** | **Function and Impact in ovarian cancer** | **Western Blot studies** | **IHC studies** | **PCR studies** | **Immunotherapy study** | **Ref** |
| --- | --- | --- | --- | --- | --- | --- | --- | --- | --- |
| MUC1 | - Overexpression of MUC1, coupled with a loss of polarization and hypo-glycosylation, is linked to resistance to apoptosis. - MUC1's rol e extends to functioning as an oncogenic molecule involved in signaling pathways that regulate apoptosis, contributing to its classification as an oncoprotein. | - Aberrant expression of MUC1 in various carcinomas is associated with poor prognosis. It is implicated in chemoresistance and influences cancer stem cell properties. - MUC1 impacts the mucosal barrier to infection, regulating innate immune responses, which ties its dysregulation to the progression of chronic epithelial diseases and cancers. | - There is ongoing research and clinical trials exploring MUC1 as a target for immunotherapy in breast cancer (BRCA) and other hematopoietic cancers like acute myeloid leukemia and myeloma. - The role of MUC1 in modulating inflammation and maintaining mucosal barrier integrity is being examined to develop targeted treatments to reduce the severity of chronic inflammatory diseases. | - MUC1 (Mucin 1 cell surface associated) is a highly glycosylated protein that is overexpressed in malignant transformations, including ovarian cancer. - Overexpression and abnormal glycosylation patterns of MUC1 are associated with resistance to apoptosis, contributing to tumor development, progression, and chemoresistance. | MUC1 protein levels have been extensively analyzed in cancer versus normal tissues, showing elevated expression in cancers, influencing apoptosis and cellular adhesion pathways. | MUC1 overexpression in various cancer tissues, establishing it as a biomarker for poor prognosis in ovarian cancer. | MUC1 mRNA levels have been quantified in cancer cell lines, correlating increased expression with cancer aggressiveness and potential therapeutic targets. | As a target for immunotherapy, with several active Phase I/II CAR T-cell trials in progress for solid and hematopoietic malignancies like leukemia and myeloma. | [96–103] |
| SORT1 | - SORT1 is elevated in the tumor microenvironment of several cancers, including ovarian, breast, prostate, colon, pancreas, skin, and pituitary cancers. - Its overexpression is related to poorer clinical outcomes, more invasive phenotypes, and increased resistance to drugs. | Targeted therapy strategies against SORT1-positive cancers, such as ovarian and breast cancers, are being developed. For instance, TH1902, a drug currently in phase 1 trials, has shown tumor suppressive effects in SORT1-positive TNBC xenograft models. | SORT1-targeted antibody-drug conjugates (ADCs) have shown effectiveness in cytotoxicity against SORT1-positive breast tumor cell lines, suggesting SORT1 as a potential target for ADCs. | Knocking down SORT1 expression in ovarian carcinoma cells resulted in increased induction of apoptosis and inhibition of proliferation. | Western blotting techniques were used to analyze SORT1 expression after RNA interference, which showed that siRNA-mediated SORT1 silencing led to reduced cancer cell proliferation, demonstrating SORT1's role in cellular proliferation. | SORT1 is more expressed in cancer tissues, especially in poorly differentiated tumors compared to adjacent non-cancerous tissues. | Real-time quantitative RT-PCR was used to quantify SORT1 transcript levels, further highlighting its significant overexpression in cancer tissues and its potential as a therapeutic target. | - SORT1 has been targeted in recent therapeutic strategies, particularly in ovarian and breast cancers. The development of antibody-drug conjugates (ADCs) targeting SORT1 has shown promise in preclinical models, with SORT1-targeted ADCs demonstrating effective cytotoxicity against SORT1-positive breast tumor cell lines. - The peptide-drug conjugate TH1902 drug shows better efficacy while targeting SORT1 than docetaxel with equivalence doses in vivo. | [73,104–109] |
| RACGAP1 | - RACGAP1 is suggested to be associated with poor overall survival (OS) and progression-free survival (PFS) in ovarian cancer, emphasizing its potential as a prognostic biomarker. - It has been shown to correlate with poor prognosis, higher proliferative phenotypes, autophagy suppression, EMT, and aggressive tumor behavior in multiple cancers. | - High RACGAP1 expression is associated with the advancement of several cancers, including colorectal, ovarian, and hepatocellular carcinoma. - It has been implicated in promoting proliferation through the AKT/CDK2 and GSK3β/Cyclin D1 pathways and metastasis via the GSK3β/β-catenin pathway. | - RACGAP1 is considered a promising therapeutic target, with the need for further exploration in malignant cancers, particularly in developing effective targeted therapies. - New strategies like antibody-drug conjugates and targeted therapies are under development and clinical trials, aiming at RACGAP1-positive tumors for potential treatment options. | RACGAP1 is overexpressed in numerous tissues and linked to poor overall survival (OS) and progression-free survival (PFS) in ovarian cancer, but not necessarily post-progression survival (PPS). | Western blot analysis confirms the presence of RACGAP1 in cancer cells, demonstrating its role in tumor biology, particularly in promoting proliferation, migration, and invasion. | Immunohistochemistry shows elevated RACGAP1 expression in various tumor tissues, including ovarian and colorectal cancers, correlating with a more aggressive tumor phenotype and worse prognosis. | Quantitative real-time PCR (qRT-PCR) assesses RACGAP1 mRNA levels, reinforcing its significant expression in tumor cells and its potential as a target for therapy. | Ongoing research investigates RACGAP1 as a prognostic and therapeutic target, with findings suggesting its involvement in crucial signaling pathways like PI3K/AKT that regulate cancer cell behavior. Its role in cancer stemness and metastatic capacity is also noted, indicating potential for targeted therapeutic strategies. | [110–115] |
| PRAME | PRAME is upregulated in epithelial ovarian cancer at both mRNA and protein levels due to the demethylation of the PRAME gene, with its DNA methylation level inversely related to expression. | PRAME is associated with poor prognosis in various cancers such as uveal melanoma and is linked with genetic changes like extra copies of chromosome 8q. Its specific immunotherapy is considered promising in treating malignancies. | PRAME is being evaluated as an immunotherapy target. Treatment with DNA methyltransferase (DNMT) inhibitors may augment PRAME immunotherapy. | PRAME expression and hypomethylation related to LIME-1 hypomethylation and lead to DNA hypomethylation in ovarian cancer | PRAME protein expression has been studied via Western blotting in cell lines and tissues, highlighting its presence in cancerous compared to non-cancerous cells. | Immunohistochemistry has shown PRAME to be more expressed in cancer tissues, especially poorly differentiated tumors compared to adjacent non-cancerous tissue. | Quantitative Real-Time PCR (qRT-PCR) techniques have been utilized to quantify PRAME mRNA expression, indicating higher levels in cancer cells. | PRAME is being explored as a target for immunotherapy. Current research includes developing T-cell receptor-based therapies and using DNA methyltransferase (DNMT) inhibitors to enhance the immunogenicity of PRAME in ovarian cancer. Such strategies are promising for treating PRAME-expressing cancers and might be effective in reversing resistance to traditional treatments like cisplatin. | [70,71,116–118] |
| UBE2C | - UBE2C is associated with a higher proliferative phenotype and histopathological grade in brain cancer, along with suppression of autophagy and apoptosis, EMT, invasion, migration, and dissemination. - High levels of UBE2C have been linked to poor patient prognosis and drug resistance, particularly in ovarian cancer. | - UBE2C has been found to be overexpressed in ovarian cancer, as confirmed by IHC studies and TCGA database analysis. It also plays a role in chemoresistance by interacting with CDK1 in ovarian tumorigenesis. - It has been proposed as a potential therapeutic target, given its crucial role in the progression of brain cancer and its impact on the cell cycle and tumor malignancy. | - UBE2C is considered a promising therapeutic target due to its interaction with the PI3K/AKT/mTOR signaling pathway, which is involved in tumor progression and metastasis. - The challenge lies in developing specific inhibitors due to the structure of E2 enzymes and the need for drugs to cross the blood-brain barrier. | UBE2C is significantly overexpressed in ovarian cancer and is linked with poor prognosis. It is associated with enhanced cell proliferation, suppression of apoptosis, promotion of epithelial-to-mesenchymal transition (EMT), invasion, migration, and drug resistance. | Western blot analysis has confirmed the overexpression of UBE2C in ovarian cancer tissues compared to normal tissues, with UBE2C being involved in pathways such as PI3K/AKT/mTOR signaling, which are critical for tumor progression and metastasis. | Immunohistochemical analysis further supports the overexpression of UBE2C in ovarian cancer tissues, highlighting its role in tumor aggressiveness and potential as a therapeutic target. | Quantitative RT-PCR has been utilized to measure UBE2C mRNA levels, reinforcing its overexpression in cancer tissues and the correlation with aggressive cancer phenotypes. | Research has identified UBE2C as a promising therapeutic target. Challenges remain in developing specific inhibitors due to the structural complexity of the enzyme and its role in crucial cellular processes. The interaction of UBE2C with key signaling pathways offers potential targets for novel therapeutic interventions. | [75,119,120] |
| EPCAM | - EpCAM is extensively expressed in various epithelial cancers and plays a crucial role in the epithelial-to-mesenchymal transition (EMT), a fundamental process in cancer metastasis. - Its expression is dynamically regulated by factors like extracellular signal-regulated kinases (ERK) and EMT transcription factors, and through regulated intramembrane proteolysis (RIP). | - - EpCAM acts as a prognostic marker, therapeutic target, and an anchor molecule on circulating and disseminated tumor cells (CTCs/DTCs). - The intracellular domain of EpCAM (EpICD) can translocate to the nucleus and interacts with proteins such as β-catenin to promote gene transcription that supports growth, cancer stem cell properties, and EMT. | - - EpCAM is targeted in various immunotherapies including monoclonal antibodies and vaccines, although the clinical outcomes have been variable. - Ongoing research is investigating its role further in EMT, which could unveil new therapeutic opportunities, particularly in managing metastasis and treatment resistance. | EPCAM is highly expressed in epithelial cancers, including ovarian cancer. Its expression during the epithelial-to-mesenchymal transition (EMT) is dynamically regulated, which impacts tumor biology in a multifaceted way. Its role in EMT, tumor growth, cancer stem cell properties, and treatment responses is complex and cancer-type dependent. | Western blotting has been used to investigate EPCAM expression in various cell types, comparing cancerous to normal cells to identify differential expression levels. | Immunohistochemical staining has been performed on tumor tissues, highlighting EPCAM as a marker for epithelial status and metastatic capacity. Variability in staining intensity and area across different studies reflects the heterogeneity of EPCAM expression in cancer. | Quantitative RT-PCR has been utilized to measure EPCAM expression at the mRNA level, with the findings suggesting a role for EPCAM in therapy resistance. | EPCAM has been a focus for immunotherapy, with various antibodies and vaccines being tested in clinical trials. Despite its long history as a therapeutic target, the effectiveness of monoclonal antibody therapy against EPCAM has been mixed. | [63,121–126] |
| CLDN3 | - The expression levels of CLDN3 in colorectal cancer (CRC) vary, with studies showing upregulation, downregulation, or no significant change. - Expression mechanisms involve DNA methylation and histone acetylation affecting gene regulation. High levels of CLDN3 correlate with low DNA methylation and high histone acetylation. - The CLDN3 promoter includes sites critical for its activity and is regulated by epigenetic factors and transcription factors like Sp1. | - - Overexpression of CLDN3 enhances cellular proliferation and invasiveness in specific models, while its downregulation is linked to conditions like pregnancy-induced hypertension. - Immunotherapy research has focused on CLDN3 as a potential biomarker and therapeutic target, leading to the development of specific antibodies. | Antibodies against CLDN3 show promise in targeting CLDN3-expressing cancer cells for therapeutic applications, including targeted cancer treatments. | CLDN3 (Claudin 3) is upregulated in various cancers, including ovarian, breast, laryngeal, and intestinal-type gastric cancers. Its expression in colorectal cancer (CRC) is controversial, with studies divided on whether it is upregulated or downregulated. | CLDN3 protein expression has been observed in normal colon tissue, but the expression levels in CRC samples vary, with some studies reporting upregulation and others reporting downregulation or stable levels. | Immunohistochemistry has shown a wide range of positive staining for CLDN3 in CRC samples, and there's inconsistency in the reports regarding its expression in normal colon and correlation with CRC grade. | Quantitative RT-PCR confirmed a correlation between DNA methylation and CLDN3 expression, with low expression levels associated with promoter methylation. | CLDN3 has been identified as a potential biomarker and therapeutic target due to its external exposure during tumorigenesis. Development of monoclonal antibodies targeting CLDN3 suggests applications in cancer diagnosis, antibody-drug conjugates (ADCs), and possibly chimeric antigen receptor (CAR) therapies for CLDN3-expressing carcinomas. | [49,127–131] |
| CXCR4 | - CXCR4 is involved in various signaling pathways that regulate cell migration, proliferation, and the dynamics within the tumor microenvironment. It also plays roles in hematopoiesis and cell homing to the bone marrow. - It mediates critical interactions necessary for inflammation, lymphoid microenvironments formation, organogenesis, and is also a major receptor for HIV-1 which complicates its functions in immune deficiency and AIDS dementia. | - - The receptor's expression is linked to the metastasis potential and severity of ovarian cancer, spotlighting it in therapeutic research. - Antagonists targeting the CXCR4/CXCL12 axis have demonstrated encouraging results, underlining its role in cancer initiation and progression. | - - CXCR4's ubiquitous expression and pivotal roles in disease mechanisms make it a valuable target for drug development. - Research indicates that downregulating CXCR4 can increase the effectiveness of chemotherapy agents such as paclitaxel via the PI3K/Akt/mTOR pathway. | CXCR4 is essential for metastasis in ovarian cancer, potentially through modulation of the Wnt/β-catenin pathway. It's identified as a significant factor in cancer cell migration, proliferation, and hematopoiesis. CXCR4's interaction with its ligand, CXCL12 (SDF-1), is pivotal in cell homing and retention in the bone marrow, and it is a major receptor for HIV-1 strains that evolve during AIDS progression. | Proteins were isolated from cells, and CXCR4 expression was analyzed using specific antibodies to quantify its level in various cancer cell types, demonstrating its ubiquitous expression and involvement in cancer cell functions. | Immunohistochemistry on paraffin-embedded samples showed CXCR4 expression in various cancers, highlighting its role in tumor cell proliferation and migration. Scoring for CXCR4 involved evaluating the intensity and frequency of staining, correlating with cancer severity. | Quantitative reverse transcriptase PCR (qRT-PCR) was utilized to measure CXCR4 mRNA levels, emphasizing its elevated expression in cancer tissues compared to normal tissues and its role in enhancing sensitivity to chemotherapy, such as paclitaxel, through pathways like PI3K/Akt/mTOR. | CXCR4's involvement in the tumor microenvironment (TME) affects immune responses, particularly through its interactions with CXCL12. This axis is crucial for tumor immunotherapy as CXCR4 antagonists have shown promising anti-cancer activities by targeting signaling pathways critical for tumor progression and regulating cancer stem cells. | [54,132–137] |
| JUP | - JUP has been implicated in multiple cancers, including melanoma, ovarian carcinoma, and non-small cell lung cancer (NSCLC). Its expression affects tumor behavior and can influence patient prognosis. - In NSCLC, high levels of JUP correlate with increased CD8 T-cell infiltration, suggesting a better clinical outcome, which contrasts with its effect in melanoma and ovarian carcinoma where its overexpression leads to decreased CD8 T-cell infiltration. | - - JUP serves as a potential prognostic marker in oral squamous cell carcinoma, promoting proliferation, migration, and invasion. - It plays a significant role in the epithelial-to-mesenchymal transition (EMT), impacting tumor grade and clinical outcomes. | - - Further research into JUP's role across various cancers could lead to targeted therapies that manipulate its expression to modulate tumor progression and immune evasion. - - The link between JUP expression and immune cell infiltration in tumors offers potential targets for immunotherapy, particularly in boosting immune responses against tumors. | JUP high expression is related and becoming a marker for ovarian cancer malignancies rather than benign. | Western blotting has been used to detect JUP expression in cell lysates, confirming its presence in cancer cells and its role in cancer biology, particularly in promoting proliferation, migration, and invasion in oral squamous cell carcinoma. | Immunohistochemical staining of JUP has shown varying expression levels across different cancers, with its localization at cell membranes indicating its role in cell adhesion and signaling pathways. | Quantitative real-time PCR (qPCR) has been used to measure JUP mRNA levels, illustrating how gene expression varies under different treatment conditions and correlating with changes in cancer cell behavior. | JUP's role in cancer makes it a target for novel therapeutic strategies. Research indicates that JUP influences melanoma growth and angiogenesis, suggesting its potential as a therapeutic target. The JUP/AGR2/LYPD3 signaling axis is particularly noted for maintaining melanoma cell stemness and mediating an immunosuppressive tumor microenvironment. | [66,138–142] |

References

96. Dhar P, McAuley J. The Role of the Cell Surface Mucin MUC1 as a Barrier to Infection and Regulator of Inflammation. Front Cell Infect Microbiol. 2019; 9:117.

<https://doi.org/10.3389/fcimb.2019.00117> PMID:[31069176](https://pubmed.ncbi.nlm.nih.gov/31069176)

97. Jin W, Liao X, Lv Y, Pang Z, Wang Y, Li Q, Liao Y, Ye Q, Chen G, Zhao K, Huang L. MUC1 induces acquired chemoresistance by upregulating ABCB1 in EGFR-dependent manner. Cell Death Dis. 2017; 8:e2980.

<https://doi.org/10.1038/cddis.2017.378> PMID:[28796259](https://pubmed.ncbi.nlm.nih.gov/28796259)

98. Li P, Xiao LY, Tan H. Muc-1 promotes migration and invasion of oral squamous cell carcinoma cells via PI3K-Akt signaling. Int J Clin Exp Pathol. 2015; 8:10365–74.

PMID:[26617744](https://pubmed.ncbi.nlm.nih.gov/26617744)

99. Li Q, Chu Y, Li S, Yu L, Deng H, Liao C, Liao X, Yang C, Qi M, Cheng J, Chen G, Huang L. The oncoprotein MUC1 facilitates breast cancer progression by promoting Pink1-dependent mitophagy via ATAD3A destabilization. Cell Death Dis. 2022; 13:899.

<https://doi.org/10.1038/s41419-022-05345-z> PMID:[36289190](https://pubmed.ncbi.nlm.nih.gov/36289190)

100. Li Z, Yang D, Guo T, Lin M. Advances in MUC1-Mediated Breast Cancer Immunotherapy. Biomolecules. 2022; 12:952.

<https://doi.org/10.3390/biom12070952> PMID:[35883508](https://pubmed.ncbi.nlm.nih.gov/35883508)

101. Supruniuk K, Radziejewska I. MUC1 is an oncoprotein with a significant role in apoptosis (Review). Int J Oncol. 2021; 59:68.

<https://doi.org/10.3892/ijo.2021.5248> PMID:[34278474](https://pubmed.ncbi.nlm.nih.gov/34278474)

102. Taylor-Papadimitriou J, Burchell JM, Graham R, Beatson R. Latest developments in MUC1 immunotherapy. Biochem Soc Trans. 2018; 46:659–68.

<https://doi.org/10.1042/BST20170400> PMID:[29784646](https://pubmed.ncbi.nlm.nih.gov/29784646)

103. Zong ZH, Du YP, Guan X, Chen S, Zhao Y. CircWHSC1 promotes ovarian cancer progression by regulating MUC1 and hTERT through sponging miR-145 and miR-1182. J Exp Clin Cancer Res. 2019; 38:437.

<https://doi.org/10.1186/s13046-019-1437-z> PMID:[31666098](https://pubmed.ncbi.nlm.nih.gov/31666098)

104. Charfi C, Demeule M, Currie JC, Larocque A, Zgheib A, Danalache BA, Ouanouki A, Béliveau R, Marsolais C, Annabi B. New Peptide-Drug Conjugates for Precise Targeting of SORT1-Mediated Vasculogenic Mimicry in the Tumor Microenvironment of TNBC-Derived MDA-MB-231 Breast and Ovarian ES-2 Clear Cell Carcinoma Cells. Front Oncol. 2021; 11:760787.

<https://doi.org/10.3389/fonc.2021.760787> PMID:[34751242](https://pubmed.ncbi.nlm.nih.gov/34751242)

105. Gao Y, Li Y, Song Z, Jin Z, Li X, Yuan C. Sortilin 1 Promotes Hepatocellular Carcinoma Cell Proliferation and Migration by Regulating Immune Cell Infiltration. J Oncol. 2022; 2022:6509028.

<https://doi.org/10.1155/2022/6509028> PMID:[35847356](https://pubmed.ncbi.nlm.nih.gov/35847356)

106. Ghaemimanesh F, Ahmadian G, Talebi S, Zarnani AH, Behmanesh M, Hemmati S, Hadavi R, Jeddi-Tehrani M, Farzi M, Akhondi MM, Rabbani H. The effect of sortilin silencing on ovarian carcinoma cells. Avicenna J Med Biotechnol. 2014; 6:169–77.

PMID:[25215181](https://pubmed.ncbi.nlm.nih.gov/25215181)

107. Simonian M, Haji Ghaffari M, Salimi A, Mirzadegan E, Sadeghi N, Ebrahimnezhad N, Fazli G, Fatemi R, Bayat AA, Milani S, Negahdari B, Rabbani H. Monoclonal Antibody Against Sortilin Induces Apoptosis in Human Breast Cancer Cells. Avicenna J Med Biotechnol. 2022; 14:37–45.

<https://doi.org/10.18502/ajmb.v14i1.8168> PMID:[35509360](https://pubmed.ncbi.nlm.nih.gov/35509360)

108. Zhuang W, Zhang W, Xie L, Wang L, Li Y, Wang Z, Zhang A, Qiu H, Feng J, Zhang B, Hu Y. Generation and Characterization of SORT1-Targeted Antibody-Drug Conjugate for the Treatment of SORT1-Positive Breast Tumor. Int J Mol Sci. 2023; 24:17631.

<https://doi.org/10.3390/ijms242417631> PMID:[38139459](https://pubmed.ncbi.nlm.nih.gov/38139459)

109. Demeule M, Charfi C, Currie JC, Larocque A, Zgheib A, Kozelko S, Béliveau R, Marsolais C, Annabi B. TH1902, a new docetaxel-peptide conjugate for the treatment of sortilin-positive triple-negative breast cancer. Cancer Sci. 2021; 112:4317–34.

<https://doi.org/10.1111/cas.15086> PMID:[34314556](https://pubmed.ncbi.nlm.nih.gov/34314556)

110. Chen S, Hu S, Zhou B, Cheng B, Tong H, Su D, Li X, Chen Y, Zhang G. Telomere-related prognostic biomarkers for survival assessments in pancreatic cancer. Sci Rep. 2023; 13:10586.

<https://doi.org/10.1038/s41598-023-37836-0> PMID:[37391503](https://pubmed.ncbi.nlm.nih.gov/37391503)

111. Eid RA, Soltan MA, Eldeen MA, Shati AA, Dawood SA, Eissa M, Zaki MSA, Algahtani M, Theyab A, Abdel-Daim MM, Kim B. Assessment of RACGAP1 as a Prognostic and Immunological Biomarker in Multiple Human Tumors: A Multiomics Analysis. Int J Mol Sci. 2022; 23:14102.

<https://doi.org/10.3390/ijms232214102> PMID:[36430577](https://pubmed.ncbi.nlm.nih.gov/36430577)

112. Gu Y, Chen B, Guo D, Pan L, Luo X, Tang J, Yang W, Zhang Y, Zhang L, Huang J, Duan R, Wang Z. Up-Regulation of RACGAP1 Promotes Progressions of Hepatocellular Carcinoma Regulated by GABPA via PI3K/AKT Pathway. Oxid Med Cell Longev. 2022; 2022:3034150.

<https://doi.org/10.1155/2022/3034150> PMID:[35958019](https://pubmed.ncbi.nlm.nih.gov/35958019)

113. Jia B, Jiang Y, Huan Y, Han Y, Liu W, Liu X, Wang Y, He L, Cao Z, He X, Zhang K, Gu J, Guo Q, Fei Z. Rac GTPase activating protein 1 promotes the glioma growth by regulating the expression of MCM3. Transl Oncol. 2023; 37:101756.

<https://doi.org/10.1016/j.tranon.2023.101756> PMID:[37595394](https://pubmed.ncbi.nlm.nih.gov/37595394)

114. Mahmoodi Chalbatani G, Gharagouzloo E, Malekraeisi MA, Azizi P, Ebrahimi A, Hamblin MR, Mahmoodzadeh H, Elkord E, Miri SR, Sanati MH, Panahi B. The integrative multi-omics approach identifies the novel competing endogenous RNA (ceRNA) network in colorectal cancer. Sci Rep. 2023; 13:19454.

<https://doi.org/10.1038/s41598-023-46620-z> PMID:[37945594](https://pubmed.ncbi.nlm.nih.gov/37945594)

115. Zhang T, Wang C, Wang K, Liang Y, Liu T, Feng L, Yang X. RacGAP1 promotes the malignant progression of cervical cancer by regulating AP-1 via miR-192 and p-JNK. Cell Death Dis. 2022; 13:604.

<https://doi.org/10.1038/s41419-022-05036-9> PMID:[35831303](https://pubmed.ncbi.nlm.nih.gov/35831303)

116. Chen X, Jiang M, Zhou S, Chen H, Song G, Wu Y, Zhu X. PRAME Promotes Cervical Cancer Proliferation and Migration via Wnt/β-Catenin Pathway Regulation. Cancers (Basel). 2023; 15:1801.

<https://doi.org/10.3390/cancers15061801> PMID:[36980687](https://pubmed.ncbi.nlm.nih.gov/36980687)

117. Gelmi MC, Gezgin G, van der Velden PA, Luyten GPM, Luk SJ, Heemskerk MHM, Jager MJ. PRAME Expression: A Target for Cancer Immunotherapy and a Prognostic Factor in Uveal Melanoma. Invest Ophthalmol Vis Sci. 2023; 64:36.

<https://doi.org/10.1167/iovs.64.15.36> PMID:[38149971](https://pubmed.ncbi.nlm.nih.gov/38149971)

118. Xu Y, Zou R, Wang J, Wang ZW, Zhu X. The role of the cancer testis antigen PRAME in tumorigenesis and immunotherapy in human cancer. Cell Prolif. 2020; 53:e12770.

<https://doi.org/10.1111/cpr.12770> PMID:[32022332](https://pubmed.ncbi.nlm.nih.gov/32022332)

119. Cai D, Tian F, Wu M, Tu J, Wang Y. UBE2C is a diagnosis and therapeutic biomarker involved in immune infiltration of cancers including lung adenocarcinoma. J Cancer. 2024; 15:1701–17.

<https://doi.org/10.7150/jca.92473> PMID:[38370368](https://pubmed.ncbi.nlm.nih.gov/38370368)

120. Domentean S, Paisana E, Cascão R, Faria CC. Role of UBE2C in Brain Cancer Invasion and Dissemination. Int J Mol Sci. 2023; 24:15792.

<https://doi.org/10.3390/ijms242115792> PMID:[37958776](https://pubmed.ncbi.nlm.nih.gov/37958776)

121. Brown TC, Sankpal NV, Gillanders WE. Functional Implications of the Dynamic Regulation of EpCAM during Epithelial-to-Mesenchymal Transition. Biomolecules. 2021; 11:956.

<https://doi.org/10.3390/biom11070956> PMID:[34209658](https://pubmed.ncbi.nlm.nih.gov/34209658)

122. Chaudry MA, Sales K, Ruf P, Lindhofer H, Winslet MC. EpCAM an immunotherapeutic target for gastrointestinal malignancy: current experience and future challenges. Br J Cancer. 2007; 96:1013–19.

<https://doi.org/10.1038/sj.bjc.6603505> PMID:[17325709](https://pubmed.ncbi.nlm.nih.gov/17325709)

123. Fagotto F, Aslemarz A. EpCAM cellular functions in adhesion and migration, and potential impact on invasion: A critical review. Biochim Biophys Acta Rev Cancer. 2020; 1874:188436.

<https://doi.org/10.1016/j.bbcan.2020.188436> PMID:[32976980](https://pubmed.ncbi.nlm.nih.gov/32976980)

124. Gires O, Pan M, Schinke H, Canis M, Baeuerle PA. Expression and function of epithelial cell adhesion molecule EpCAM: where are we after 40 years? Cancer Metastasis Rev. 2020; 39:969–87.

<https://doi.org/10.1007/s10555-020-09898-3> PMID:[32507912](https://pubmed.ncbi.nlm.nih.gov/32507912)

125. Macdonald J, Henri J, Roy K, Hays E, Bauer M, Veedu RN, Pouliot N, Shigdar S. EpCAM Immunotherapy versus Specific Targeted Delivery of Drugs. Cancers (Basel). 2018; 10:19.

<https://doi.org/10.3390/cancers10010019> PMID:[29329202](https://pubmed.ncbi.nlm.nih.gov/29329202)

126. Sun X, Martin RCG, Zheng Q, Farmer R, Pandit H, Li X, Jacob K, Suo J, Li Y. Drug-induced expression of EpCAM contributes to therapy resistance in esophageal adenocarcinoma. Cell Oncol (Dordr). 2018; 41:651–62.

<https://doi.org/10.1007/s13402-018-0399-z> PMID:[30116994](https://pubmed.ncbi.nlm.nih.gov/30116994)

127. Bürgel N, Bojarski C, Mankertz J, Zeitz M, Fromm M, Schulzke JD. Mechanisms of diarrhea in collagenous colitis. Gastroenterology. 2002; 123:433–43.

<https://doi.org/10.1053/gast.2002.34784> PMID:[12145796](https://pubmed.ncbi.nlm.nih.gov/12145796)

128. Cox KE, Liu S, Hoffman RM, Batra SK, Dhawan P, Bouvet M. The Expression of the Claudin Family of Proteins in Colorectal Cancer. Biomolecules. 2024; 14:272.

<https://doi.org/10.3390/biom14030272> PMID:[38540693](https://pubmed.ncbi.nlm.nih.gov/38540693)

129. Orea MJ, Angulo JC, González-Corpas A, Echegaray D, Marvá M, Lobo MVT, Colás B, Ropero S. Claudin-3 Loss of Expression Is a Prognostic Marker in Castration-Resistant Prostate Cancer. Int J Mol Sci. 2023; 24:803.

<https://doi.org/10.3390/ijms24010803> PMID:[36614243](https://pubmed.ncbi.nlm.nih.gov/36614243)

130. Yang H, Park H, Lee YJ, Choi JY, Kim T, Rajasekaran N, Lee S, Song K, Hong S, Choi JS, Shim H, Kim YD, Hwang S, et al. Development of Human Monoclonal Antibody for Claudin-3 Overexpressing Carcinoma Targeting. Biomolecules. 2019; 10:51.

<https://doi.org/10.3390/biom10010051> PMID:[31905631](https://pubmed.ncbi.nlm.nih.gov/31905631)

131. Zhao A, Qi Y, Liu K. CLDN3 expression and function in pregnancy-induced hypertension. Exp Ther Med. 2020; 20:3798–806.

<https://doi.org/10.3892/etm.2020.9084> PMID:[32855729](https://pubmed.ncbi.nlm.nih.gov/32855729)

132. Figueras A, Alsina-Sanchís E, Lahiguera Á, Abreu M, Muinelo-Romay L, Moreno-Bueno G, Casanovas O, Graupera M, Matias-Guiu X, Vidal A, Villanueva A, Viñals F. A Role for CXCR4 in Peritoneal and Hematogenous Ovarian Cancer Dissemination. Mol Cancer Ther. 2018; 17:532–43.

<https://doi.org/10.1158/1535-7163.MCT-17-0643> PMID:[29146630](https://pubmed.ncbi.nlm.nih.gov/29146630)

133. Mezzapelle R, Leo M, Caprioglio F, Colley LS, Lamarca A, Sabatino L, Colantuoni V, Crippa MP, Bianchi ME. CXCR4/CXCL12 Activities in the Tumor Microenvironment and Implications for Tumor Immunotherapy. Cancers (Basel). 2022; 14:2314.

<https://doi.org/10.3390/cancers14092314> PMID:[35565443](https://pubmed.ncbi.nlm.nih.gov/35565443)

134. Wang J, Cai J, Han F, Yang C, Tong Q, Cao T, Wu L, Wang Z. Silencing of CXCR4 blocks progression of ovarian cancer and depresses canonical Wnt signaling pathway. Int J Gynecol Cancer. 2011; 21:981–7.

<https://doi.org/10.1097/IGC.0b013e31821d2543> PMID:[21738044](https://pubmed.ncbi.nlm.nih.gov/21738044)

135. Zi D, Li Q, Xu CX, Zhou ZW, Song GB, Hu CB, Wen F, Yang HL, Nie L, Zhao X, Tan J, Zhou SF, He ZX. CXCR4 knockdown enhances sensitivity of paclitaxel via the PI3K/Akt/mTOR pathway in ovarian carcinoma. Aging (Albany NY). 2022; 14:4673–98.

<https://doi.org/10.18632/aging.203241> PMID:[35681259](https://pubmed.ncbi.nlm.nih.gov/35681259)

136. Zhou W, Guo S, Liu M, Burow ME, Wang G. Targeting CXCL12/CXCR4 Axis in Tumor Immunotherapy. Curr Med Chem. 2019; 26:3026–41.

<https://doi.org/10.2174/0929867324666170830111531> PMID:[28875842](https://pubmed.ncbi.nlm.nih.gov/28875842)

137. Zou YR, Kottmann AH, Kuroda M, Taniuchi I, Littman DR. Function of the chemokine receptor CXCR4 in haematopoiesis and in cerebellar development. Nature. 1998; 393:595–9.

<https://doi.org/10.1038/31269>
PMID:[9634238](https://pubmed.ncbi.nlm.nih.gov/9634238)

138. Chae YK, Choi WM, Bae WH, Anker J, Davis AA, Agte S, Iams WT, Cruz M, Matsangou M, Giles FJ. Overexpression of adhesion molecules and barrier molecules is associated with differential infiltration of immune cells in non-small cell lung cancer. Sci Rep. 2018; 8:1023.

<https://doi.org/10.1038/s41598-018-19454-3> PMID:[29348685](https://pubmed.ncbi.nlm.nih.gov/29348685)

139. Fang J, Xiao L, Zhang Q, Peng Y, Wang Z, Liu Y. Junction plakoglobin, a potential prognostic marker of oral squamous cell carcinoma, promotes proliferation, migration and invasion. J Oral Pathol Med. 2020; 49:30–8.

<https://doi.org/10.1111/jop.12952> PMID:[31420988](https://pubmed.ncbi.nlm.nih.gov/31420988)

140. Hu YD, Wu K, Liu YJ, Zhang Q, Shen H, Ji J, Fang D, Xi SY. LY6/PLAUR domain containing 3 (LYPD3) maintains melanoma cell stemness and mediates an immunosuppressive microenvironment. Biol Direct. 2023; 18:72.

<https://doi.org/10.1186/s13062-023-00424-3> PMID:[37924160](https://pubmed.ncbi.nlm.nih.gov/37924160)

141. Leick KM, Rodriguez AB, Melssen MM, Benamar M, Lindsay RS, Eki R, Du KP, Parlak M, Abbas T, Engelhard VH, Slingluff CL Jr. The Barrier Molecules Junction Plakoglobin, Filaggrin, and Dystonin Play Roles in Melanoma Growth and Angiogenesis. Ann Surg. 2019; 270:712–22.

<https://doi.org/10.1097/SLA.0000000000003522> PMID:[31425296](https://pubmed.ncbi.nlm.nih.gov/31425296)

142. Spethmann T, Böckelmann LC, Labitzky V, Ahlers AK, Schröder-Schwarz J, Bonk S, Simon R, Sauter G, Huland H, Kypta R, Schumacher U, Lange T. Opposing prognostic relevance of junction plakoglobin in distinct prostate cancer patient subsets. Mol Oncol. 2021; 15:1956–69.

<https://doi.org/10.1002/1878-0261.12922> PMID:[33533127](https://pubmed.ncbi.nlm.nih.gov/33533127)
