# Supplementary material for: Discovery of differentially expressed proteins for CAR-T therapy of ovarian cancers with a bioinformatics analysis
Source: Aging (Albany NY). 2024 Jul 18;16(14):11409–33. doi: 10.18632/aging.206024 (PMC11315388; doi:10.18632/aging.206024)
Supplement: Supplementary Table 2 [file aging-16-206024-s003.pdf]

SUPPLEMENTARY TABLES

Supplementary Table 2. RT-PCR primer on EpCAM, MUC-1, and PRAME in ovarian cancer based on Kloudová et al., (2016) study.

Name of marker (protein)	FOR primer (5´-3´)	REV primer (5´-3´)	Probe (5´-3´)	No. of ovarian cancer samples examined	Positive result (%)
EpCAM	GCAGGGTCTAA AAGCTGGTGT	ACCCATCTCCTTT ATCTCAGCCTT	TGCTGTTATTGTGGT TGTGGTGATAGCAGT	41	>90%
MUC-1	CGTAGCCCCTA TGAGAAGGTTTC	GCGACGTGCCC CTACAAG	AGCAGCCTCTCTTAC ACAAACCCAGCA	41	>90%
PRAME	CGTTTGTGGGG TTCCATTC	CCAGAGGGAG GCAGGTG	TGGCTGTGTCTCCCG TCAAAGGC	41	>60%
